# Visualizing atomic-scale redox dynamics in vanadium oxide-based catalysts

**DOI:** 10.1038/s41467-017-00385-y

**Published:** 2017-08-21

**Authors:** Martin Ek, Quentin M. Ramasse, Logi Arnarson, Poul Georg Moses, Stig Helveg

**Affiliations:** 1 Haldor Topsøe A/S, Haldor Topsøes Allé 1, 2800 Kgs Lyngby, Denmark; 2 SuperSTEM Laboratory, STFC Daresbury, Keckwick Lane, Daresbury, WA4 4AD UK

## Abstract

Surface redox processes involving oxygen atom exchange are fundamental in catalytic reactions mediated by metal oxides. These processes are often difficult to uncover due to changes in the surface stoichiometry and atomic arrangement. Here we employ high-resolution transmission electron microscopy to study vanadium oxide supported on titanium dioxide, which is of relevance as a catalyst in, e.g., nitrogen oxide emission abatement for environmental protection. The observations reveal a reversible transformation of the vanadium oxide surface between an ordered and disordered state, concomitant with a reversible change in the vanadium oxidation state, when alternating between oxidizing and reducing conditions. The transformation depends on the anatase titanium dioxide surface termination and the vanadium oxide layer thickness, suggesting that the properties of vanadium oxide are sensitive to the supporting oxide. These atomic-resolution observations offer a basis for rationalizing previous reports on shape-sensitive catalytic properties.

## Abstract (web summary)

Redox processes in metal oxide surfaces can exhibit structure sensitivities which are difficult to uncover. Here, the authors use atomic-resolution imaging to demonstrate facet dependent alterations in the surfaces of supported vanadium oxide upon reduction and oxidation.

## Introduction

Oxide materials play a significant role in heterogeneous catalysis^1, 2^. In their own right, oxides can facilitate the conversion of molecular species. They are also employed as carrier materials to disperse active elements into nanometer-sized particles that enhance the availability of surface sites. The reactivity of oxides is often attributed to coordinatively unsaturated sites, which are proposed to be oxygen-deficient defects that enable reactions involving lattice oxygen atoms^3–7^. Atomic-scale information about surface sites in oxide catalyst materials is, however, challenging to retrieve, as oxide surfaces can undergo substantial reconstructions and exchange oxygen atoms with the surrounding atmosphere in ways that are difficult to predict^8, 9^.

One illustrative example of such a complex behavior is provided by vanadium oxide supported on titanium dioxide (VO_x_/TiO_2_). The VO_x_/TiO_2_ system can catalyze a variety of chemical reactions, including the selective reduction of NO_x_ for emission abatement from power plants and diesel engines^10, 11^, and oxidation and ammoxidation of various organic substrates^12, 13^. These diverse catalytic properties of the TiO_2_-supported VO_x_ have been subject to much debate. Some studies have suggested a dependence on the TiO_2_ crystal structure (e.g., rutile, anatase, or TiO_2_-B)^12, 14, 15^ and even on the surface faceting^16, 17^. These findings are contradicted by other studies which explicitly report an insensitivity to the support structure^11, 13, 18, 19^. In addition, the surrounding gas environment^12, 13^ and the thickness of the VO_x_ layer^12–14, 20^ are also considered to have an influence. A coherent understanding of these features of the catalyst surface has not yet been established. One approach to address the structure sensitivity has been to investigate extended single crystal surfaces as model systems by using surface sensitive techniques^8, 9^. Such studies do indicate the existence of support effects^21, 22^. However, relating this insight to the technical catalysts is difficult because the model and technical catalysts are prepared by different synthesis procedures and use TiO_2_ supports with different crystal structures. Hence, observations of technical VO_x_/TiO_2_ catalysts, made at atomic resolution and under relevant reaction conditions, should be beneficial for extending the understanding of their properties and functions.

Transmission electron microscopy (TEM) has emerged as a powerful tool for observing surface structures of oxides ex situ, particularly so for nanostructured materials^23–25^. The introduction of gas cells has furthermore enabled numerous in situ studies of metal catalyst surfaces under reactive gas environments^26, 27^. Similar observations of oxide catalyst materials are comparatively scarce^28, 29^ and have mainly been used for the study of bulk oxidation and reduction^30, 31^, including bulk-structured V_2_O_5_^32^. However, the crystal structure of supported VO_x_ can be influenced by the underlying oxide^14–17, 21, 22^ and the broken translational symmetry at the surface. The redox processes for supported VO_x_ might therefore differ from those in the corresponding bulk materials.

Here we use atomic-resolution TEM in combination with electron energy loss spectroscopy (EELS) to examine the surface structure and oxidation state of VO_x_, supported on anatase TiO_2_ nanoparticles, in situ, under alternating oxidizing and reducing environments. Our observations reveal that the outermost atomic layer of the VO_x_/TiO_2_ catalyst undergoes a reversible order-disorder structural transformation, concomitant with a change in the V oxidation state, when changing the oxidation potential of the reactive gas environment. As the technical catalyst is based on nanoparticles with multiple surface terminations, the observations directly reveal that surface redox dynamics is sensitive to the structure of the supporting TiO_2_ facet. The present observations therefore provide a basis for rationalizing previous reports on shape-sensitive catalytic properties of VO_x_/TiO_2_, and for interpreting findings from single–crystal model catalyst studies in a coherent way.

## Results

### Vanadium oxide distribution

In order to relate the redox properties of the vanadium oxide layer with its thickness, two samples of anatase TiO_2_ nanoparticles were prepared with nominal VO_x_ loadings of 2 and 0.5 monolayers. One monolayer corresponds to 7–8 V/nm^2^, matching the Ti atom density at the anatase (001) surface. Raman spectroscopy^13^ confirmed that the overall VO_x_ loadings were in the few- and sub-monolayer regime, respectively, as detailed in Supplementary Fig. 1. Prior to the in situ experiments we determined the distribution of VO_x_ over the TiO_2_ nanoparticles using ex situ scanning TEM (STEM) and EELS.

**Fig. 1:**
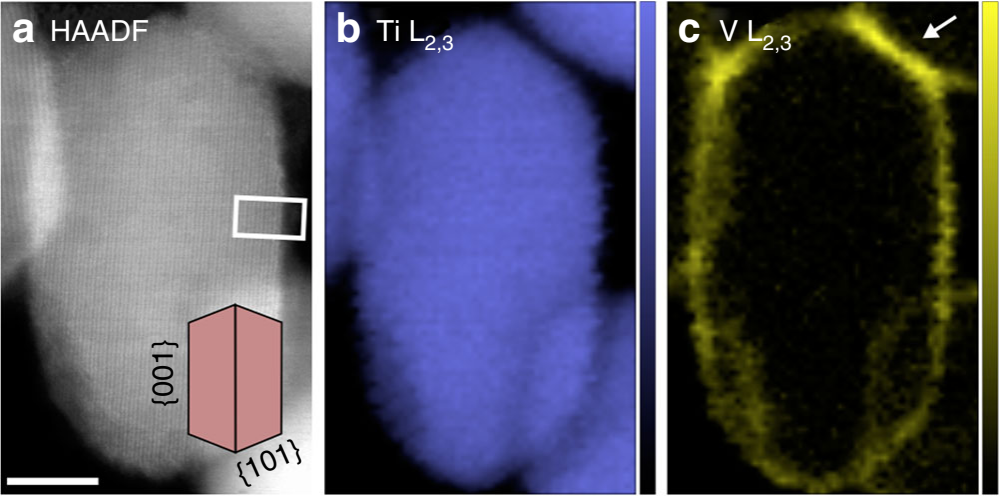
Vanadium distribution over a single anatase TiO_2_ nanoparticle. **a** HAADF image of a VO_x_/TiO_2_ particle imaged in a [010] direction with an inset model illustrating the faceting. An EEL SI recorded from the same area was used to generate the elemental maps in **b**, **c** which show the intensity of the L_2,3_ edge of Ti (*color scale* 0–5300 counts) and V (*color scale* 0–1400 counts), respectively. The *white arrow* indicates an area at the junction between two particles with a small excess of vanadium. *Scale bar*, 5 nm

Figure 1a shows one high angle annular dark-field (HAADF) STEM image of a 2 monolayer VO_x_/TiO_2_ particle. The image was recorded in a [010] orientation which advantageously aligns the major anatase facet types, i.e., {001} and {101}, parallel to the electron beam, as illustrated by the inset model. Supplementary Fig. 2 shows the same particle at higher magnification to allow verification of its orientation and crystal structure. Only the low index {001} and {101} were present as extended surface terminations on this particle, comprising about 35% and 65%, respectively, of the total surface area. This morphology was confirmed to be representative of the TiO_2_ raw material by powder X-ray diffraction (XRD), which measured average diameters of 24 and 39 nm in the ⟨001⟩ and ⟨101⟩ directions, respectively. For the ⟨100⟩ direction, the average diameter was measured to be 42 nm, which is too large for the corresponding {100} surface termination to be present, in full agreement with the electron microscopy characterization. No traces of other TiO_2_ polymorphs, e.g. rutile, were detected by XRD (see Supplementary Fig. 3).

An electron energy loss (EEL) spectrum image (SI) was subsequently recorded from the same area and used to construct elemental maps. Figure 1b, c shows the distribution of titanium and vanadium over the particle. Details on how electron-beam-induced changes during the SI acquisition were evaluated and mitigated are given in Supplementary Fig. 2. The SI processing at the Ti and V L_2,3_ ionization edges to yield the elemental maps is illustrated in Supplementary Fig. 4. Figure 1c shows that the V L_2,3_ map has similar intensity around the perimeter of the particle, indicating that the two dominating surface facets of the anatase particle contain similar amounts of vanadium oxides. The increased vanadium signal at the perimeter of the particle, as compared to the center, is attributed to these surfaces being parallel to the electron beam, leading to a higher number of vanadium atoms in the electron beam direction. The V L_2,3_ maps from the 0.5 monolayer sample showed peak intensities at the particle perimeters roughly one quarter of those from the 2 monolayer sample, i.e., 110–180 compared to 400–600 counts/pixel, and therefore indicate an even distribution of vanadium oxides over the TiO_2_ surface also for the lower coverage. Several additional examples from both samples are given in Supplementary Fig. 5. No areas were ever found to be completely depleted of vanadium oxides, even for the 0.5 monolayer sample. The elemental maps, however, show the sum of the vanadium signal projected in the viewing direction. As the surfaces extend for several nanometers, an even distribution of vanadium oxides will give the appearance of a continuous coverage. The very even distribution indicates a strong interaction between the supporting TiO_2_ and the vanadium oxide surface phase, allowing it to spread out over the support particles.

Occasionally, the impregnation resulted in small excesses of vanadium oxides at the concave areas where two or more particles were in contact. This can be seen for instance in the top right corner of Fig. 1c, marked with a white arrow, where the vanadium signal is greater than the sum at the facets away from the junction area. Such excess areas were also found in the 0.5 monolayer sample. Likely, the excess of vanadium oxides found at the junction was caused by capillary forces attracting more impregnation liquid than the free facets during the drying process. There is, however, no indication from the STEM images that vanadium oxide in these excess areas differs in structure from the vanadium oxide on the free facets, e.g., by having formed a segregated bulk V_2_O_5_ phase. Raman spectroscopy confirmed the absence of bulk V_2_O_5_ in the 0.5 monolayer sample (Supplementary Fig. 1). Therefore, the similar looking excess areas in the 2 monolayer sample likely do not consist of bulk V_2_O_5_ either. The trace amounts of bulk V_2_O_5_ that were found in the 2 monolayer sample by Raman spectroscopy might instead be located elsewhere in the sample.

**Fig. 2:**
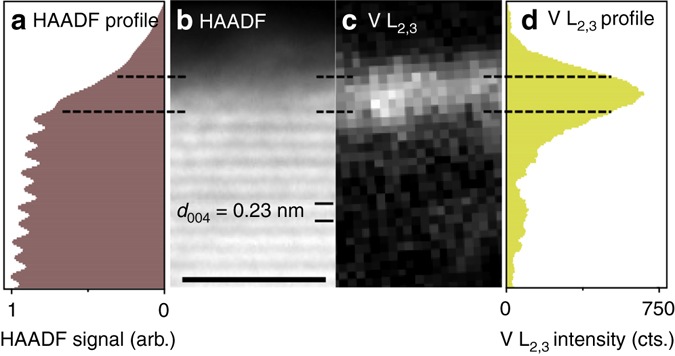
Profile of the vanadium distribution on a TiO_2_ (001) surface. HAADF intensity profile **a** and corresponding image **b** together with an EELS V L_2,3_ map **c** and profile **d** recorded at the same area, showing the distribution of vanadium on a small area of the (001) facet indicated in Fig. 1a. The peak in the V L_2,3_ profile occurs on top of the outermost (004) crystal plane seen in the HAADF image and profile. The *dashed lines* indicate a layer on the surface with a thickness of two (004) planes, placed symmetrically around the peak in the V L_2,3_ profile, which contains the outermost crystalline layer and some amorphous material on top. *Scale bar*, 2 nm

Figure 2 shows a V L_2,3_ map from the area on the (001) surface marked in Fig. 1a acquired at higher spatial sampling, together with the corresponding HAADF image. The vanadium signal in the V L_2,3_ map peaks on top of the outermost (004) crystal plane in the HAADF image, which can be seen from the two profiles in Fig. 2a, d. The position and width of the vanadium signal peak indicates that the particle was covered with vanadium oxides in excess of a single monolayer in thickness at this location, but not exceeding 2 monolayers, consistent with its nominal 2 monolayer loading. The exact thickness of the vanadium oxide layer is, however, difficult to measure as the surfaces of the anatase nanoparticles are not perfectly flat. The dashed lines indicate a distance of two anatase (004) layers, positioned symmetrically around the peak in the vanadium profile, which contains the outermost crystalline layer of the particle as well as some amorphous material on top. On top of this amorphous VO_x_ layer, a second amorphous phase with lower HAADF intensity can be seen. This second phase is attributed to carbonaceous material built up over time under illumination by the electron beam and did not affect the elemental analysis. A more detailed characterization of the structure of the surface VO_x_ layers was performed using in situ TEM as discussed in the coming sections.

### Vanadium oxidation state

The detailed fine structure of the vanadium L-edge in the EEL spectra reflects the local chemical bonding environment around the excited atoms and therefore provides information about the vanadium oxidation state. Specifically, the energy loss associated with the V L_3_ peak is reported to shift ~2.5 eV over the range of VO to V_2_O_5_ in bulk oxides, providing a simple measure of the vanadium oxidation state^33^. For the present catalysts, the measured peak positions from different agglomerates varied by less than 0.2 eV and 0.5 eV for the 2 and 0.5 monolayer samples, respectively. Therefore, the EELS measurements provide adequate precision for distinguishing the different VO_x_ oxidation states, even for catalysts with sub-monolayer loadings.

For the STEM-EELS acquisitions, the V L_3_ peak position remained constant when recording consecutive SIs of the same area, indicating that the electron beam did not continuously reduce the vanadium oxide. Comparisons between the STEM-EEL spectra and EEL spectra from bulk vanadium oxides–from comparisons with previous EELS^33^ and XPS^34^ reports–indicate a closer resemblance to V_2_O_3_ than to V_2_O_5_. The state of the supported vanadium oxide in the microscope vacuum therefore differed from the V^5+^ state of the as-synthesized sample, and of the state under catalytically relevant conditions^13^. To address this apparent gap in oxidation state, the combined effects of an oxygen atmosphere and elevated temperatures were explored in situ by EEL spectroscopy.

**Fig. 3:**
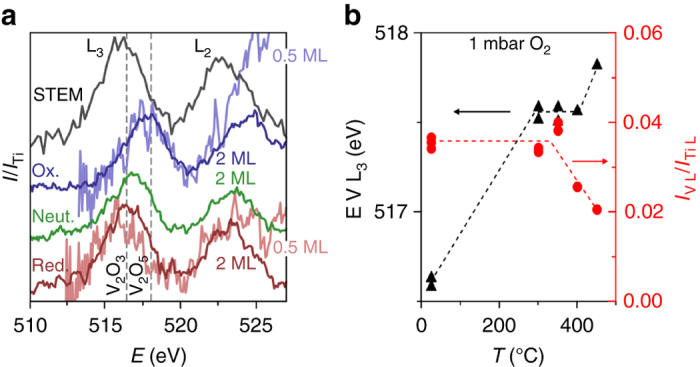
In situ measurements of the vanadium oxidation state by EELS. **a** Vanadium L_2,3_-edge region of EEL spectra recorded from both 2 and 0.5 monolayer VO_x_/TiO_2_ particles, normalized to the Ti L_2,3_ edge intensity. For the 0.5 monolayer sample, the signal has been multiplied by four in order to show the spectra from the two samples on the same intensity scale. For both samples, the V L-edge shows a marked shift in energy loss as the conditions were changed in situ from neutral (room temperature, vacuum), to reducing (300 °C, vacuum), and to oxidizing (300 °C, 1 mbar O_2_) conditions. A spectrum extracted from a STEM SI is included for comparison. The energy losses of the V L_3_ peak for two bulk vanadium oxides^33^ are also indicated. **b** Position and normalized height of the V L_3_ peak, measured by fitting a Gaussian function to background subtracted EEL spectra, obtained during heating of the 2 monolayer sample in 1 mbar O_2_. At 300 °C, a plateau was reached with a stable vanadium oxidation state. Above ca. 450 °C, approximately half of the 2 monolayers of vanadium oxide was lost, accompanied by a small further oxidation. The difference of < 0.2 eV of the V L_3_-peak position between multiple measurements illustrates the high precision of the EELS measurement

Immediately after inserting the sample into the base vacuum of the in situ electron microscope, EEL spectra from agglomerates of both 2 a﻿nd 0.5 monolayer VO_x_/TiO_2_ particles showed the vanadium oxide as partially reduced. That is, Fig. 3a shows that in this “neutral” state, the V L_3_ peak is positioned between that of bulk V_2_O_5_ and V_2_O_3_, meaning that the VO_x_ did not reduce all the way to VO_1.5_ as in the STEM instrument. The small difference in the initial degree of reduction measured in the two instruments might be due to differences in base vacuum, time from insertion to measurement, or sample pre-conditioning procedure (which included an overnight bake-out step for the STEM instrument, see Methods section for details). Heating the VO_x_/TiO_2_ particles to 300 °C in the base vacuum of the in situ microscope (10^−6^ mbar) did in fact result in a slight shift of the V L_3_ peak to a lower energy loss. This provided an excellent match to bulk V_2_O_3_, and to the spectra recorded in the ultra-high vacuum (UHV) environment of the STEM instrument after the gentle bake-out. In contrast, introducing 1 mbar O_2_ at the sample region resulted in a shift of the V L_3_ peak by ~1.6 eV from 516.0–516.4 eV to 517.5–518.0 eV, indicating an oxidation of vanadium to closely match bulk V_2_O_5_. Figure 3b shows that this full oxidation of the 2 monolayer VO_x_/TiO_2_ sample was reached at 300 °C with no further changes occurring up to 400 °C. This temperature interval is also consistent with the catalytic conditions for VO_x_/TiO_2_ in for instance selective catalytic reduction^11^ and partial oxidations^12^. The shifts of the V L_3_ peak with gas atmosphere at 300 °C were fully reversible and could be observed over several oxidation and reduction cycles (Supplementary Fig. 6), and occurred with an unchanged intensity of the V L-edge, corresponding to a constant coverage of vanadium atoms. It was thereby possible to control the oxidation state of the vanadium oxide in situ, allowing for detailed structural characterization of both the partially reduced VO_1.5_ state, found at the high vacuum conditions, and the pristine VO_2.5_, representative of the active state of the catalyst, as described in the next section. In addition, heating of VO_x_/TiO_2_ in H_2_ and CO environments was also examined as detailed in Supplementary Fig. 7. Specifically, the H_2_ treatment resulted in the formation of VO_2_, and CO treatment resulted in VO_1.5-2_. These oxidation states are included in the range covered by the reduction and oxidation under high vacuum and O_2_ exposure, respectively. Therefore we will focus on the latter conditions in order to address the relation between the structural and chemical state over the full range of reversible oxidation states.

Heating the 2 monolayer sample above ~450 °C in oxygen led to decreasing V L_3_ peak intensities and possibly a small further shift of the V L_3_ peak to higher energy losses, as shown by Fig. 3b. The corresponding EEL spectra are shown in Supplementary Fig. 8. Interestingly, the intensity was roughly halved before stabilizing, indicating that roughly half of the vanadium oxide was lost from the 2 monolayer VO_x_/TiO_2_ particles and that the remaining ca. 1 monolayer was more resilient. The higher thermal stability of the first monolayer is further evidence of the strong interaction between the vanadium oxide and supporting TiO_2_. The vanadium signal did not recover upon subsequent reduction and oxidation at 300 °C, but the remaining vanadium oxide exhibited similar shifts of the V L_3_ edge as the original 2 monolayer sample before the high temperature oxidation (Supplementary Fig. 8).

In addition to the temperature and atmosphere, the vanadium oxidation state was also affected by the electron illumination. To evaluate this effect, EEL spectra were recorded from VO_x_/TiO_2_ agglomerates at varying electron illumination (Supplementary Fig. 9). With the samples kept in vacuum at room temperature, which was found to be the most electron-beam-sensitive condition, the vanadium L_2,3_-edge remained constant until the electron dose rate exceeded 300 e^−^Å^−2^s^−1^. Below this dose rate, illuminating the sample continuously even for an extended period of 15 min had negligible effect on spectral features, indicating that the accumulated electron dose was not perturbing the samples. However, at higher electron dose rates the V L_3_ peak shifted to lower energy losses, indicating an electron-dose-rate stimulated reduction of the vanadium oxide. All spectra and images presented here were therefore acquired with an electron dose rate of 300 e^−^Å^−2^s^−1^ (images) or below (spectra), and can therefore be considered unaffected by the electron beam.

### Structural changes with reduction/oxidation

In order to determine whether the change in oxidation state coincides with any differences in crystal structure or distribution of material at the surfaces of the oxide particles, high-resolution TEM images were recorded from the same VO_x_/TiO_2_ particles under both the reducing and oxidizing conditions. The low dose-rate imaging conditions allowed long-time observations of the same particle without the formation of reduced VO particles or any other detectable structural changes (Supplementary Fig. 10), in accordance with the limits measured by EELS. Due to the rather low electron dose rate, individual TEM images had a low signal-to-noise ratio. Series of consecutive images were therefore acquired at varying focus and combined by means of exit-wave (EW) reconstruction to improve the signal-to-noise ratio and to correct for residual aberrations in the electron optics^35^. The phase of such reconstructed exit waves (referred to as EW phase images) contains intensity maxima which coincide in position with the atomic columns^36^. Specifically, the cation columns (V or Ti) provide the most prominent features in the images while the lighter oxygen columns are mainly visible as gray bands (Supplementary Methods). Due to the small difference in atomic number between Ti and V a quantitative assessment of the atomic content is difficult. However, the element mapping (Fig. 1) unambiguously shows that all free surfaces of the TiO_2_ particles were evenly covered with vanadium oxides. Due to drift induced by changes in temperature or gas environment the nanoparticles occasionally tilted away from the intended [010] zone axis, resulting in changes to the contrast pattern. For the present study we selected nanoparticles with a near zone-axis orientation for which the relation between the phase maxima and cation columns remained (Supplementary Fig. 11).

**Fig. 4:**
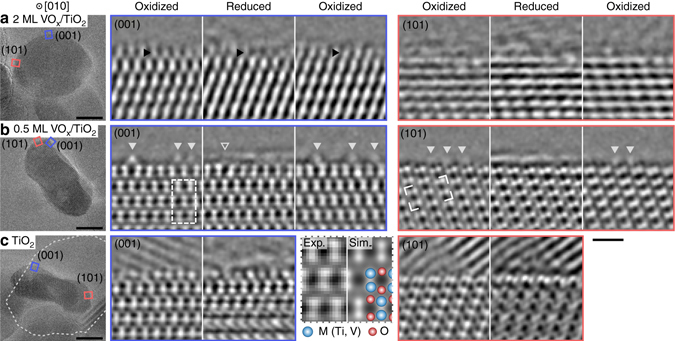
Reversible changes in the vanadium oxide surface layer imaged by TEM. **a** High-resolution TEM image of a 2 monolayer VO_x_/TiO_2_ particle indicating the areas on the (001) and (101) surfaces cropped from the subsequent EW phase images and shown throughout the reduction-oxidation cycle. The crystalline contrast of the outermost layer on the (001) surface, indicated by *black arrows*, was greatly reduced under the reducing conditions (300 °C, vacuum), but reappeared in the exact same position when O_2_ was introduced again (300 °C, 1 mbar O_2_). No such change was visible at the (101) surface. Corresponding images are also shown for a 0.5 monolayer VO_x_/TiO_2_ particle **b** and for a pure TiO_2_ particle **c**, neither of which undergoes a similar change in the structure of the outermost layer. The bright arrows in **b** indicate single surface atomic rows that shifted between equivalent anatase positions. The lattice fringes outside the particle surface in **c** stems from the larger, underlying particle outlined in the overview image, which superimpose contrast in the image over the free surfaces. The *dashed box* at the (001) surface in **b** is shown at higher magnification together with a simulated EW phase overlaid with an anatase structure model. The same pattern is also indicated at the (101) surface to show their relative orientation. *Scale bar* for the overview images, 10 nm. *Scale bar* for the EW phase images, 1 nm

By ensuring that each EW captured entire catalyst particles, it was possible to align the EW phase images recorded under oxidizing and reducing conditions by means of sub-pixel phase correlation. Using the unchanging structure of the TiO_2_ support as a reference (Supplementary Fig. 12) the alignment achieved atomic precision, allowing the comparison of the structure of the surfaces under the different conditions on an atomic column basis. As shown in Fig. 4, the morphology of the surface layer remained unaltered as the vanadium oxide was transformed between the reduced and oxidized states, implying the absence of any redistribution of material between the different surface facets. This indicates that the distribution of the reduced vanadium oxides found by STEM-EELS in UHV is representative also for the catalytically relevant, fully oxidized VO_2.5_.

In Fig. 4a, b, the anatase structure of the interior of the particles can be seen to extend all the way to the surface on both the (001) and (101) surfaces, indicating that the surface vanadium oxides are isostructural to the TiO_2_ support for both the 2 and 0.5 monolayer catalysts. The contrast of the outermost surface layer was often lower than of that immediately below, indicating that it consisted of patches of VO_x_ that only partially covered the surface. For the 2 monolayer catalyst, this observation is fully consistent with a coverage in excess of a single monolayer and below two monolayers, as found by STEM-EELS and presented in Fig. 2. In the interior of the catalyst particles, the variations in contrast in Fig. 4 between the atomic layers are much smaller and may be attributed to variation in the projected thickness.

Careful examination of the (001) facet on the 2 monolayer sample in Fig. 4a reveals that the contrast from the outermost layer of metal atoms, observed under oxidizing conditions, was reduced and smeared under the reducing conditions, but reappeared at the same position after re-establishing the oxidizing conditions. For the (101) facet, no changes were detected during the change in conditions. This distinct, qualitative contrast difference between the two surface configurations forms the basis for the following analysis. The reversible alteration of the outermost surface layer was observed for all studied 2 monolayer VO_x_/TiO_2_ particles, but not at all for the 0.5 monolayer VO_x_/TiO_2_ or for pure TiO_2_ particles under identical conditions, as shown in Fig. 4b, c. Further examples are given in Supplementary Figs. 13–16, showing two additional particles from separate TEM specimens for the 2 and 0.5 monolayer catalysts and one additional particle for pure TiO_2_. The specificity of the reversible alteration indicates that only the second layer of vanadium atoms was involved in the surface transformation and that the properties of the VO_x_ surface phase were influenced by the structure of the supporting anatase surface. In addition to the reversible alteration, single rows of atoms on the surfaces were occasionally displaced between equivalent positions, as shown in Fig. 4b. The displacements of single atomic rows, however, occurred on both the (001) and (101) facets, suggesting they are intrinsic to all anatase surfaces. While an effect of the environment cannot be excluded, these displacements are very similar to the previously reported movement of single atomic rows on the surfaces of pure TiO_2_, which is a thermally activated process^29^.

The reduced contrast at the (001) surface of the 2 monolayer VO_x_/TiO_2_ particles indicates that vanadium atoms lost their isostructural positions at the anatase particle surface. Since the process was fully reversible, and no significant morphological change of the catalyst particles was observed, the VO_x_ cannot have been fully removed from the surface, but must instead have been rearranged such that its contribution to the TEM image contrast was smeared out. The smearing could result from an increased mobility or a disordering of the vanadium atoms on the surface, giving the appearance of an amorphous layer with a faint contrast, as in Fig. 4a. In cases where the initial crystalline contrast of the outermost layer was low, such smearing diminished the contrast to the point where it could no longer be detected in the image, giving the appearance that it disappeared (e.g., Supplementary Fig. 13a). The amorphous layer seen under the reducing conditions in Fig. 4b, however, likely originated from a monolayer of adsorbed contaminants. Such contamination layers can easily be distinguished from the disordering of the outermost vanadium atoms: the former adds on top of the outermost crystalline layer while the latter evolves from a transformation of this crystalline surface layer (Supplementary Note 1). Furthermore, the contamination layers diminished with repeated redox cycles with no effect on the reversible structural alteration of the surface VO_x_. The proposal that the reduced vanadium oxide remained on the anatase surfaces, albeit in a mobile or disordered state, is further supported by the STEM observations in Fig. 2, which show that the reduced vanadium oxide extended beyond the outermost visible crystalline layer.

**Fig. 5:**
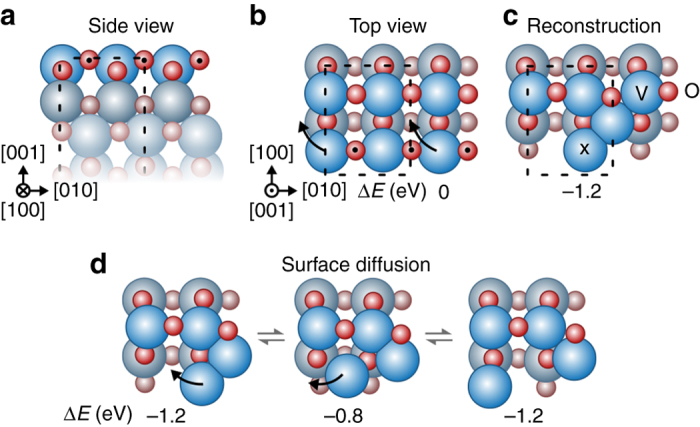
Structure and surface diffusion of a reduced VO_x_ layer. Structure of a 2 monolayer VO_2_ phase isostructural to a fixed anatase (001) surface viewed **a** *perpendicular* and **b** *normal* to the surface. Only the two surface layers are shown for the top views in **b**–**d**. The twofolded oxygen atoms marked with *black dots* were removed to reduce the surface layer, which could subsequently reconstruct by shifting the under-coordinated vanadium atoms according to the arrows to form the VO_1.5_ layer in **c**. The unit cell for the reduced phase is indicated by a dashed box in **a**–**c**. **d** The path between two equivalent sites in the unit cell for a single vanadium atom, denoted with ‘x’ in **c**, is shown by arrows in the models. The transition state constitutes a barrier of only 0.4 eV. The arbitrary choice of which oxygen atoms were removed controlled which of the surface vanadium atoms that exhibited the greatest mobility. However, all surface vanadium atoms are equivalent and can each be made mobile by removing the appropriate oxygen atoms. All energies are given with respect to the unreconstructed VO_1.5_ structure

To further substantiate this interpretation, density function theory (DFT) calculations were performed to address the structural response of an anatase supported vanadium oxide phase upon reduction. Figure 5a, b shows a model of a (001) surface comprising two VO_2_ monolayers attached to four fixed anatase VO_2_ layers to simulate the effect of the isostructural match with the support. The transformation to a reduced state under the high vacuum conditions was modeled by removing half of the twofold coordinated and therefore most loosely bound oxygen atoms^8^. This removal results in a VO_1.5_ composition for the outermost layer. The VO_1.5_ layer was unstable with respect to displacement of the under-coordinated vanadium atoms. Specifically, moving half of the vanadium atoms, as indicated in Fig. 5b, c, reduces the energy of the surface by 1.2 eV per shifted vanadium atom. For the surface diffusion pathway of the outermost vanadium atoms, illustrated in Fig. 5d, the DFT analysis found an energy barrier of only 0.4 eV. With this barrier, the surface vanadium atoms can undergo of the order of millions of such diffusion events during the time needed for recording a TEM image, even at room temperature. Note that the first vanadium oxide monolayer, directly attached to the fixed anatase support, remains largely unchanged throughout both the reduction and surface diffusion processes. These calculations therefore indicate a disordered structure and high mobility for the vanadium atoms in the outermost (001) layer of the reduced phase, which consistently supports the interpretation of the in situ TEM observations in Fig. 4.

Removing the twofold coordinated oxygen atoms from the (101) surface also results in a VO_1.5_ composition for the outermost layer. However, the DFT calculations show a much smaller structural response upon reduction of the (101) compared to the (001) surface, as detailed in Supplementary Fig. 17, involving only small displacements of the surface vanadium atoms. Qualitatively, this difference can be explained by the lower initial under-coordination of the vanadium atoms in the reduced (101) surface; the lowest oxygen coordination for any vanadium atom is fourfold and threefold, respectively, in the (101) and (001) surfaces (see Supplementary Figs. 17c, d and Fig. 5b with twofold coordinated O atoms removed). Furthermore, the higher density of vanadium atoms on the (101) surface than on the (001) surface will lead to higher barriers for surface diffusion.

It is furthermore informative to compare the surface transformation discussed above with that occurring at the high temperature oxidation, where EEL spectroscopy indicated that half of the 2 monolayer VO_x_ had been removed. Supplementary Fig. 18 shows the outermost VO_x_ layer on a (001) facet of a 2 monolayer VO_x_/TiO_2_ particle first going through the reversible transformation at 300 °C, and subsequently being removed after a high temperature oxidation step. Together with the previously discussed EEL measurements and high-resolution images (Figs. 3 and 4), this observation indicates that the reversible surface transformation was contingent on the vanadium atoms in the second monolayer remaining on the particles.

### Difference in oxidizability between 0.5 and 2 monolayer vanadium oxide

**Fig. 6:**
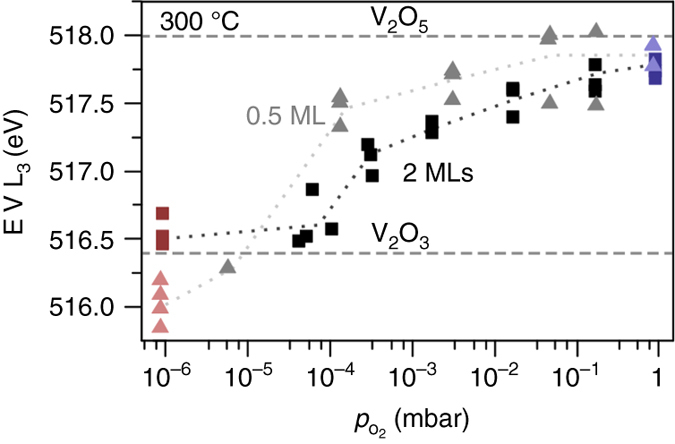
Oxidation of 2 and 0.5 monolayer vanadium oxides on TiO_2_ with increasing O_2_ pressures. Position of the vanadium L_3_ peak, measured by fitting a Gaussian function to background subtracted EEL spectra, acquired at 300 °C for increasing O_2_ pressures. The initial (~10^−6^ mbar, *red symbols*) and end states (1 mbar, *blue symbols*) correspond to the reduced and oxidized states in Fig. 3. The intermediate steps show that the oxidation occurred at lower O_2_ partial pressures for the 0.5 monolayer (*gray* ▲) than the 2 monolayer supported vanadium oxide (*black* ■)

Although the oxidation state of the 0.5 and 2 monolayer VO_x_/TiO_2_ samples under the reducing and oxidizing conditions appeared similar, as shown by Fig. 3, some subtle differences could be detected. Figure 6 shows the energy loss of the V L_3_ peak as a function of O_2_ pressure for both the 0.5 and 2 monolayer samples. In the base vacuum of the in situ microscope (*p* = ~10^−6^ mbar), corresponding to the reducing conditions, the V L_3_ energy loss was slightly lower for the 0.5 monolayer sample than for the 2 monolayer sample, indicating a higher oxygen deficiency for the former. Increasing the oxygen pressure gradually shifted the V L_3_ energy loss towards that of the bulk V_2_O_5_ reference. For the 0.5 monolayer sample, the V L_3_ energy loss rapidly increased at about 10^−4^ mbar, whereas the same shift extended over almost two orders of magnitude higher oxygen partial pressures for the 2 monolayer catalyst. At high pressures (*p*_O2_ = 1 mbar), the V L_3_ energy loss was close to that of the V_2_O_5_ reference for both samples, indicating similar oxidation states. The gradual shift of the V L_3_ peak at the intermediate steps in the oxidation process is likely due to the signal from several different vanadium oxide species being superimposed in the spectrum.

The difference between the 0.5 and 2 monolayer VO_x_/TiO_2_ samples is consistent with previous DFT calculations. Vittadini et al. have reported that the oxygen partial pressure at which the outermost vanadium oxide layer is oxidized from VO_2_ to VO_2.5_ increases with the number of vanadium oxide monolayers in the range of 1–3 monolayers for vanadium oxide supported on a (001) anatase TiO_2_ surface^37^. This trend continues below a single monolayer, as the calculated energy required for removing an oxygen atom from a 0.5 monolayer vanadium oxide surface phase is greater than for a full monolayer (shown in Supplementary Fig. 19).

## Discussion

Due to the small difference in the average atomic number of the two oxides, a combination of electron microscopy techniques is necessary to firmly relate the structural and chemical state of VO_x_/TiO_2_. Here, chemical mapping by means of STEM-EELS spectrum imaging was used to address the homogeneity of the VO_x_ distribution, while in situ TEM imaging was used to monitor the structural response to changes in the surrounding gas environment. The two-step approach was necessitated by the fundamental differences in the electron microscopy operating conditions: STEM-EELS spectrum imaging typically requires high electron dose rates to generate sufficient signal in the EEL spectra, while low dose rates are required for in situ TEM in order to supress beam induced sample alterations during repeated imaging at atomic resolution in gas environments^35, 38–41^.

Previously, ex situ electron microscopy investigations of VO_x_/TiO_2_ catalysts have often relied on the high susceptibility of VO_x_ to electron-beam-induced changes, which allows their selective reduction to VO using prolonged illumination^42^. The rock salt crystal structure type of the VO provides clear structural contrast to the supporting TiO_2_ and enables the presence of surface VO_x_ phases to be confirmed^43–45^. Supplementary Fig. 20 show that such structural alteration of the surface layers by the electron beam could be formed on the present samples as well. However, this alteration required the use of higher electron dose rates and accumulated doses than were used during the acquisition of the spectra and images presented elsewhere in this report. Thus, the present approach demonstrates that VO_x_/TiO_2_ can be examined by electron microscopy with suppression of electron-beam-induced structural alterations.

The structure of the VO_x_/TiO_2_ catalyst can be compared with previous studies of epitaxial growth of vanadium oxides on single crystal TiO_2_ anatase surfaces. Depositions using molecular beam epitaxy (MBE) on the anatase (001) surface results in epitaxial and coherent structures of V_2_O_4_ extending over several monolayers^46^. Subsequent exposure to oxygen will then oxidize the surface, forming an ordered overlayer detectable by low energy electron diffraction^46^. DFT calculations suggest that the oxidation only affects the outermost VO_x_ layer; the underlying layers remain as VO_2_ while the outermost layer forms VO_2.5_ or VO_3_ so that the overall stoichiometry approaches V_2_O_5_^37^. This is fully consistent with the present finding that the vanadium atoms remained in the anatase cation positions under the oxidizing conditions where the average oxidation state was V^5+^. As a consequence, the supported VO_2.5_ surface phase thus formed differs in structure from a simple truncation of bulk V_2_O_5_.

The in situ TEM images presented in Fig. 4 likewise indicate that the anatase structure was retained for the first monolayer of VO_x_ directly attached to the TiO_2_ support in the reduced state. For the (001) surface, the DFT calculations consistently show a minimal effect on the first VO_x_ layer after removing low-coordinated oxygen atoms from the second surface VO_x_ layer to accomplish the reduction. This retention of a VO_2_ layer can also explain why the 2 monolayer sample appeared slightly less reduced than the 0.5 monolayer sample, as shown by Fig. 6; the average composition of a VO_1.5_ surface layer and an underlying VO_2_ layer becomes VO_1.75_. On the (001) surface, there are additional low-coordinated oxygen atoms which can be removed to bring the average composition closer to VO_1.5_. However, on the (101) surface the remaining oxygen atoms are bound to three vanadium atoms and therefore less likely to be removed, preventing the average composition from completely reaching VO_1.5_.

The surface reconstruction predicted by DFT for the reduced state of the (001) facet of the 2 monolayer VO_x_/TiO_2_ catalyst was attributed to the under-coordination of the vanadium atoms after losing part of their neighboring oxygen atoms. For a corresponding reduction of bulk V_2_O_5_, the under-coordination is compensated by a coherent displacement of cations leading to crystallographic shear^30^. On the surface, however, the vanadium atom displacements are less restricted and may thereby form a disordered structure rather than a net shear. Furthermore, the reduced coordination at the surface also results in a very low barrier for the vanadium atoms to shift between equivalent sites. These surface-specific properties are crucial for understanding the in situ TEM results. According to the present findings, the disorder and mobility of the surface vanadium atoms should gradually decrease with increasing oxygen pressure as the surface oxidizes. During the gradual oxidation at 300 °C, Fig. 6 shows the emergence of VO_2_, corresponding to the V L_3_ energy loss of 517 eV, already at a pressure of ca. 2 × 10^−4^ mbar O_2_. At this point, the twofold coordinated oxygen sites on the surfaces are fully occupied. Thus the transition from the disordered state to the crystalline state occurs over a narrow range of oxygen pressures.

While anatase is the most commonly used TiO_2_ polymorph in technical catalysts, rutile predominates in single crystal model systems due to the higher availability of suitable substrates^8, 9^. Such model systems have been used to study the effects of oxidation and reduction on the structure of VO_x_ supported on rutile (110). On this surface, 2 monolayers of VO_x_ reversibly transforms between a fully isostructural state for V^4+^ to an uncorrelated state upon complete oxidation to V^5+^. Note that the transformation on rutile involves oxidation and loss of structural correlation to the support for both VO_x_ layers^21^. For the present anatase based catalyst, oxidation at 300 °C was limited to the outermost VO_x_ layer and did not affect its anatase structure. Even after the high temperature oxidation step, one monolayer remained attached and isostructural to the anatase support. The increased stability of the first VO_x_ monolayer on anatase is supported by the previously mentioned MBE growth studies; a single epitaxial V_2_O_5_ monolayer form on both (001) and (101) anatase surfaces during deposition in oxygen, while subsequent layers become disordered^46, 47^. The difference in behavior of VO_x_ on anatase and rutile indicates that the interaction between VO_x_ and TiO_2_ is strongly sensitive to the crystal structure of the support. This finding indicates the importance for model studies to emphasize TiO_2_ polymorphs and surfaces resembling those actually used in its technical applications.

Previous studies of the activity and selectivity of VO_x_/TiO_2_ catalysts have found measurable effects of the TiO_2_ crystal structure^14^ and morphology^16, 17^. However, these variations have alternatively been proposed to originate from differences in the dispersion of the VO_x_ over the particles^12^, or differences in the nature and amount of impurities remaining from the synthesis^13^. The observations of differences over single TiO_2_ particles with an even distribution of VO_x_ circumvent these objections. Thus, the present demonstration of facet-dependent redox dynamics for VO_x_ on anatase TiO_2_ may have general implications for the understanding of the catalytic properties of the VO_x_/TiO_2_ system. For instance, the dynamic behavior of the {001} facets, facilitated by the lower coordination of its surface atoms, could allow for a more efficient exposure of surface sites that favorably facilitate adsorption and conversion of reactants. Such an effect could explain why nanostructured VO_x_/TiO_2_ catalysts with a larger fraction of {001} over {101} surfaces have been reported to be more active for the selective reduction of NO by NH_3_^16^, and more reducible by H_2_^17^.

In the future, descriptions of catalytic reactions mediated by VO_x_/TiO_2_ catalysts should be advanced by including information about facet-dependent redox dynamics, in combination with reactivity data, measured or calculated for simple model systems. Thus, the ability to observe oxide catalysts at atomic resolution under meaningful chemical conditions is essential for developing atomic-scale understanding of oxide-catalyzed chemical reactions, and of redox chemistry of nanostructured oxide materials in general.

## Methods

### Synthesis

The VO_x_/TiO_2_ samples were prepared from commercially available pure anatase TiO_2_ nanoparticles (DT51, Crystal), which were impregnated with a vanadate solution using the incipient wetness method to a loading corresponding to 2.5 and 10 wt% V_2_O_5_. After drying overnight, the impregnated powders were calcined in air at 470 °C for 3 h. Reference measurements were performed on the pure TiO_2_ powder with no further treatment. Samples for in situ TEM and ex situ STEM-EELS characterization were prepared by crushing and grinding, followed by dry dispersion directly onto plasma cleaned Protochips MEMS^48^ devices and standard lacy carbon Cu TEM grids, respectively.

### STEM-EELS chemical mapping

STEM and EEL spectrum imaging was carried out at the SuperSTEM laboratory, Daresbury, using a Nion UltraSTEM100 aberration-corrected scanning transmission electron microscope operated at 60 kV. The samples were baked at 125 °C overnight in vacuum prior to loading them into the microscope. An UHV below 7 × 10^−9^ mbar was maintained at the sample area during analysis. The probe-forming optics was configured to provide a beam convergence semi-angle of 32 mrad, corresponding to a probe size of approximately 0.11 nm. EEL SIs were acquired at a sampling of typically 0.3–0.5 spectra/nm with a dwell time of 0.05 s/spectrum, a 37 mrad collection semi-angle and a nominal 0.2 eV/channel dispersion. All SIs were processed using principal component analysis to remove instrumental noise^49^. The maps shown in Figs. 1 and 2, and Supplementary Fig. 5 were produced by fitting a power-law background under the edge of interest, followed by signal integration over a 14 eV window from the edge onset (chosen to avoid any overlap between the V L and O K edges). Due to the strong background from the Ti edge, the background for the V edge was also fitted to the channels immediately before the O K edge onset. The energy shift was calibrated using the Ti L_2_ peak while the energy dispersion was calibrated using the Ti L_2_ and O K edges.

### In situ TEM

In situ TEM was carried out using a Titan ETEM operated at 300 kV with a spherical aberration coefficient (after correction) of ca. −15 µm. The instrument has a base pressure below 2 × 10^−6^ mbar when the liquid nitrogen cold trap is not in use. The microelectrome chanical systems (MEMS) heating device temperature was estimated using the factory supplied calibration in vacuum. When introducing gases (O_2_, up to 1 mbar) into the differentially-pumped sample area, the heating current was increased in order to keep the measured resistance constant, thereby compensating for the increased heat loss to the gas^50^. The electron dose rate was monitored using the factory calibration of the microscope viewing screen, and was kept below 300 e^−^Å^−2^s^−1^ at all times for the particles used for high resolution imaging. For the EW reconstructions, 35 member focal series with 2 s exposure time and 2 nm defocus step from 40 to −30 nm were acquired. Reconstructions were performed using the Gerchberg-Saxton algorithm^51^, as implemented in the MacTempas software (www.totalresolution.com). EEL spectra were acquired in diffraction mode using a 2.7 mrad collection semi-angle and a 0.1 eV/channel dispersion, typically from a micrometer sized area on an agglomerate. Power-law backgrounds were fitted prior to the Ti and V edges. The energy shift was calibrated using the Ti L_2_ peak while the energy dispersion was calibrated using the Ti L_2_ and O K edges.

### XRD

Powder samples were measured on a PANanalytical Empyrean diffractometer working in Bragg-Brentano geometry using Cu Kα radiation. Estimation of coherent domain sizes was based on the Debye-Scherrer equation using the TOPAS software employing the fundamental parameters approach. Lorentzian single peak fits on the (004), (101), and (200) reflections were used to deduce size in the respective crystallographic directions.

### DFT

DFT calculations were performed using the Grid-based Projector Augmented Wave (GPAW) program^52, 53^ with the general gradient approximation (GGA) BEEF-VdW functional^54^. The wavefunctions were represented by linear combination of atomic orbitals (LCAO) with ‘dzp’ basis set. The VO_x_/TiO_2_(001) was represented by 6 layers of VO_2_ in anatase crystal structure where the four lowest layers were fixed during the calculations to mimic the TiO_2_ support. A k-point mesh of 2 × 2 × 1 was used for sampling the primitive unit cell, which was periodic in the *xy* plane with 10 Å vacuum between successive slabs in the perpendicular direction. The nudged elastic band algorithm with the climbing image method was used to locate transition states^55^.

### Data availability

The data reported in this study are available from the corresponding author upon reasonable request.

## Electronic supplementary material

Supplementary Information
