# Supplementary material for: Visualizing atomic-scale redox dynamics in vanadium oxide-based catalysts
Source: Nat Commun. 2017 Aug 21;8:305. doi: 10.1038/s41467-017-00385-y (PMC5563508; doi:10.1038/s41467-017-00385-y)
Supplement: Supplementary file 1 — Supplementary Information [file 41467_2017_385_MOESM1_ESM.pdf]

File Name: Supplementary Information
Descriptions: Supplementary Figures, Supplementary Note, Supplementary Methods and Supplementary References

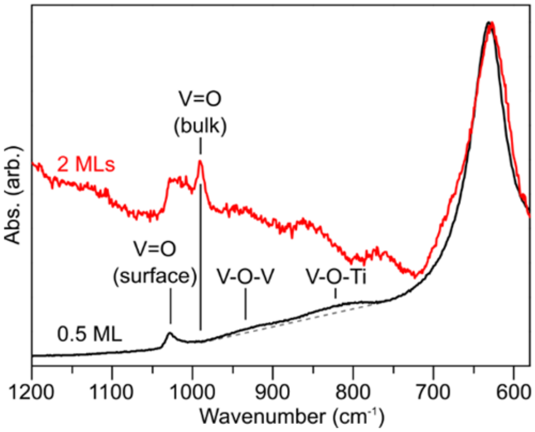

Supplementary Figure 1. Raman spectra recorded from the 0.5 and 2 monolayer VO_x/TiO_2 samples. The Raman spectra were acquired in air at 400 °C using a 633 nm He-Ne laser. The 2 monolayer sample was calcined overnight at 300 °C in dry air prior to acquiring the spectrum, since the sample exhibited significant fluorescence deteriorating the Raman spectrum. The main feature of the 0.5 monolayer sample is the V=O stretch band at 1028 cm^{-1}, which is typically observed for pure, well-dispersed and dehydrated sub-monolayer VO_x/TiO_2 samples. The two broad bands at 920 and 800 cm^{-1} are assigned to V-O-V and V-O-Ti (possibly including contributions from Ti-O-Ti) bending modes,[1] although these assignments are currently subject to debate.[2,3] The fact that both bands are present indicates that the density of the surface V-oxides is in the regime where both monomeric (bonding only to the TiO_2 surface) and polymeric (bonding also to neighbouring V) species are present. For the spectrum from the 0.5 monolayer sample, a linear background has been drawn between 1000 and 750 cm^{-1} in order to show more clearly the area beneath the two V-O-M (M= V or Ti) bands. The larger absorption by the V-O-Ti groups indicates that loading is slightly below half a monolayer. For the 2 monolayer sample the V=O band at 1028 cm^{-1} is still present. In addition a sharp peak is present at 995 cm^{-1} related to V=O stretching in bulk-like V_2O_5. The cross section for V=O stretching is however much higher for the vanadyl groups in the bulk-like V_2O_5 than for the vanadyl groups on the surface-supported species, and only a small fraction of the V-oxides exceeding the first monolayer has been incorporated into the bulk-like phase.

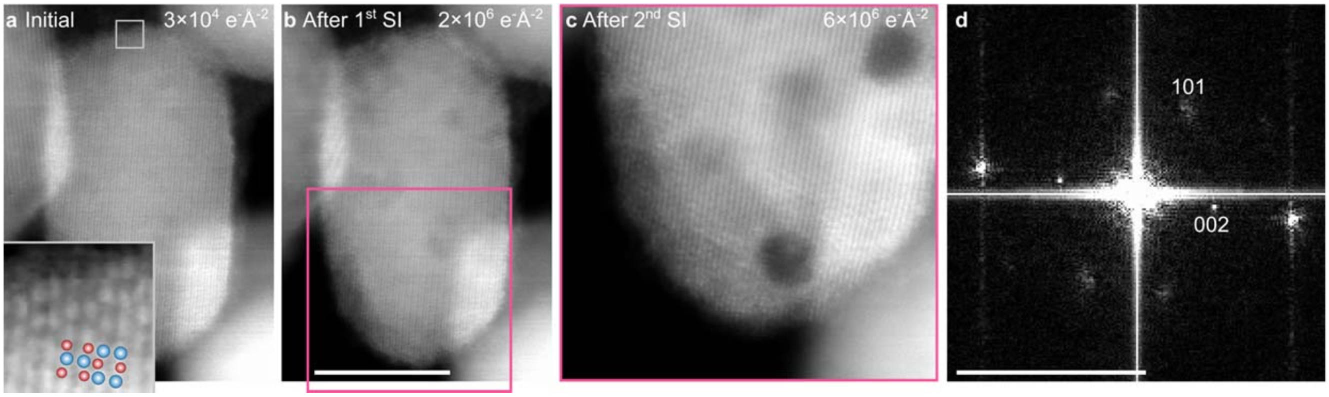

Supplementary Figure 2. Electron beam damage during the recording of EEL SIs. HAADF images recorded from the same particle shown in figures 1 and 2 in the main text (**a**) prior to the first SI, (**b**) immediately after the first SI, and (**c**) after a second SI had been recorded from the bottom half of the particle. The inset in **a** shows a detail from the particle surface, recorded with increased sampling simultaneously with the first SI. For the inset, a median filter has been applied to reduce noise. In the HAADF-STEM image, the cation columns appear with bright contrast and are located in the anatase structure all the way to the surface. After the first SI (**b**), the particle surfaces appeared unchanged, indicating that redistribution of materials during the SI acquisition was suppressed. However, a few darker spots appeared in the centre of the particle indicating the initial stages of beam damage in these regions. (**c**) In the HAADF image acquired after the second SI, the damage became more marked as seen from the more pronounced dark spots. The corresponding SI was therefore discarded. The estimated accumulated electron dose is indicated in each panel. At no point, even for the highest sampling SIs, were cubic TiO or VO phases observed to form at the particle surfaces, which has been reported as a major beam effect for both the pure oxides[4] and for VO_x/TiO_2.[5–7] This indicates that at the comparatively short beam exposure times used here, the main damage mechanism is sputtering rather than beam-induced reduction. The more marked beam effects seen for STEM compared to TEM imaging is likely related to the much higher electron dose rate of the STEM beam together with the UHV environment (cf. Supplementary Fig. 9). Scale bar, 10 nm. (**d**) FFT of the image in **c** with the 2.10 nm^{-1} (002) and the 2.84 nm^{-1} (101) reflections marked, which demonstrate the anatase structure and [010] viewing direction for the particle (scale bar, 5 nm^{-1}).

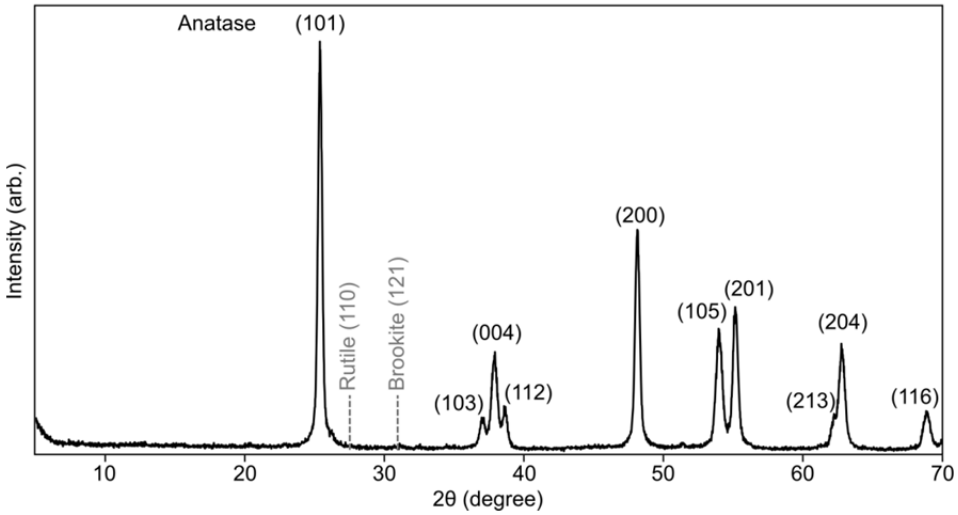

Supplementary Figure 3. Powder X-ray diffraction pattern from the 2 monolayer VO_x/TiO_2. The powder sample was measured on a PANanalytical Empyrean diffractometer working in Bragg-Brentano geometry using Cu Kα radiation. The diffraction pattern has been indexed according to the anatase structure. The absence of other TiO_2 polymorphs is evident from the fact that anatase alone can account for all peaks. The positions where the main unique peaks of rutile and brookite would have been visible are indicated to show the high degree of phase purity of the sample. Lorentzian single-peak fits on the anatase (004), (101), and (200) reflections were used to deduce average particle sizes of 24, 39 and 42 nm in the respective crystallographic directions. The resulting crystal shape – a truncated square bipyramid bound by {001} and {101} surfaces – is well represented by the nanoparticle shown in figure 1 in the main text.

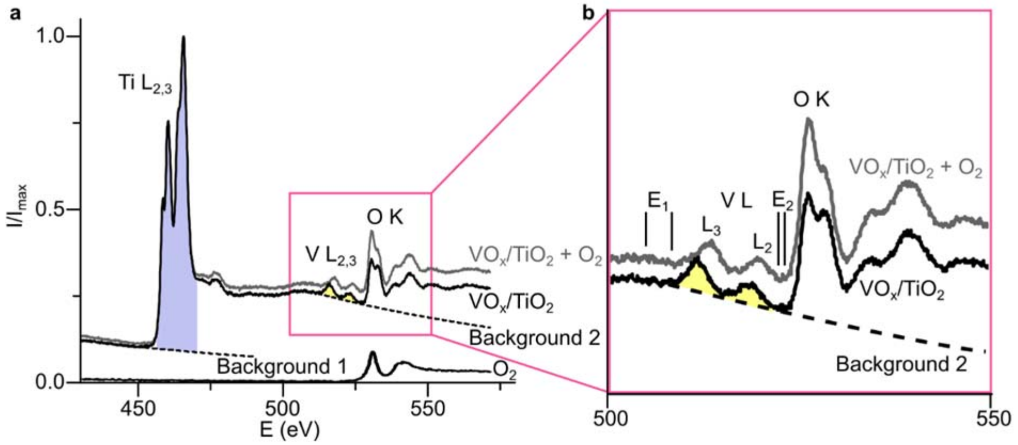

Supplementary Figure 4. Overview of typical VO_x/TiO_2 EEL spectra. (**a**) Two spectra acquired at an elevated temperature (300 °C) in vacuum and a 1 mbar O_2 atmosphere, respectively. All spectra (including those in the main text and other supplementary figures) have been normalized so that the Ti L_2 peak has a maximum intensity of 1. The spectra were aligned using the Ti $L_{2,3}$ edge, which stems mainly from the bulk of the TiO_2 particles and is not expected to change under the observation conditions applied here. It therefore provides a very stable reference which allowed the energy shift of the V edge to be measured accurately from the position of the V L_3 peak (in the range of 516-518 eV). The O K edge will also under most circumstances stem mainly from the oxygen in the bulk of the TiO_2 particle (as much fewer oxygen atoms are located in the supported vanadium oxide phase), and can therefore provide an accurate calibration of the EEL spectrometer energy dispersion. Only for measurements performed in an oxygen atmosphere was there a noticeable contribution to the O K edge which did not stem from the bulk of the TiO_2 particles. The added signal from the gas-phase oxygen did not affect the nearby vanadium edge. A spectrum recorded only from the 1 mbar O_2 background is included for comparison. The power-law backgrounds fitted prior to the Ti and V edges are shown as dashed lines. The coloured areas indicate the integration windows used to generate the maps in figures 1 and 2 in the main text. (**b**) A more detailed view of the V and O edges, indicating the two energy ranges, E_1 and E_2, used to generate the power-law background for the V $L_{2,3}$ edge and onwards to higher energy losses.

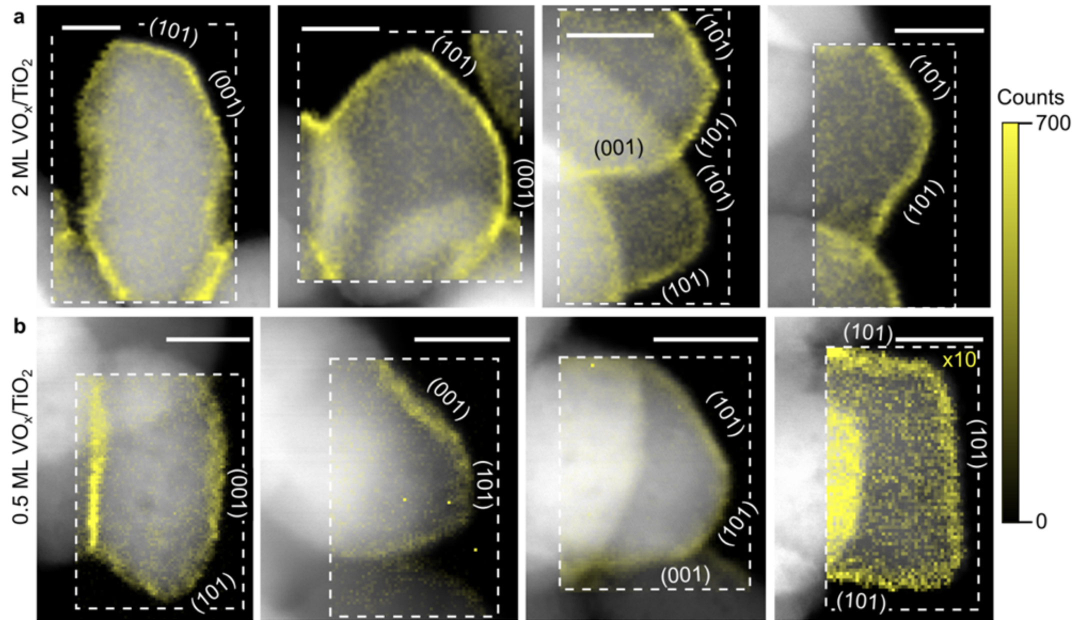

Supplementary Figure 5. Additional vanadium EELS maps. HAADF images overlaid with EELS maps of the V $L_{2,3}$ edge from the (**a**) 2 monolayer (ML) and (**b**) 0.5 monolayer VO_x/TiO_2 samples (generated using a 14 eV integration window from the edge onset). The maps are displayed with the same intensity scale going from transparent to opaque over 0-700 counts (except for the last panel in **b** where the intensity has been multiplied by 10). As the SIs were acquired with the same electron illumination parameters, the intensities can be compared between the images. The facets at the perimeter of the particles have been indexed where possible from the lattice fringes in the HAADF images or the corresponding bright-field images. The two rightmost panels in **a** and **b** show particles imaged in the [111] direction, where only {101} facets are parallel to the beam at the particle perimeter. The remaining particles were all imaged in [010], and had both {001} and {101} facets parallel to the beam (although the first particle in **a** was slightly titled so that the (001) facet was not well aligned with the beam in this case). Even though the particles were located in different parts of the agglomerates, or in different agglomerates altogether, they show very similar loadings and distributions of vanadium. There is also no indication of a difference in loading between the two major facet types. Scale bars, 10 nm.

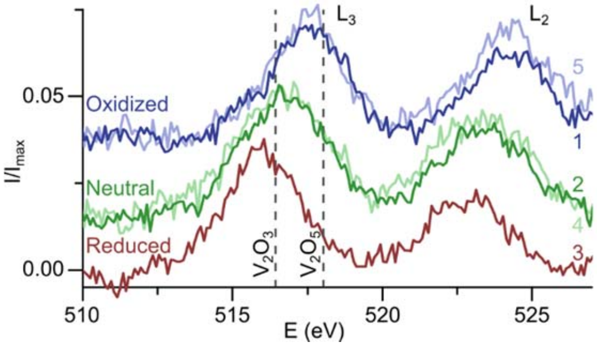

Supplementary Figure 6. EEL spectra recorded during repeated reduction/oxidation. EEL spectra recorded from a micrometre-sized area of a VO_x/TiO_2 agglomerate under oxidizing (300 °C, 1 mbar O_2), neutral (RT, vacuum 10^{-6} mbar), and reducing (300 °C, vacuum 10^{-6} mbar) conditions. All spectra are shown after subtracting a power-law background and have been normalized so that the Ti L_2 peak has a peak intensity of 1. The spectra recorded under neutral and oxidizing conditions have been vertically offset. Each spectrum is numbered according to the order in which it was acquired while changing the conditions from oxidizing to reducing via neutral, and back to oxidizing again. The reproducibility of the oxidation states of the supported vanadium oxide film can be seen by comparing the vanadium $L_{2,3}$ peaks from the first and second oxidation cycle in terms of position and intensity under both the neutral (spectrum 2 and 4) and oxidizing (spectrum 1 and 5) conditions. The L_3-peak positions for two bulk vanadium oxides[8] are indicated by dashed lines for reference.

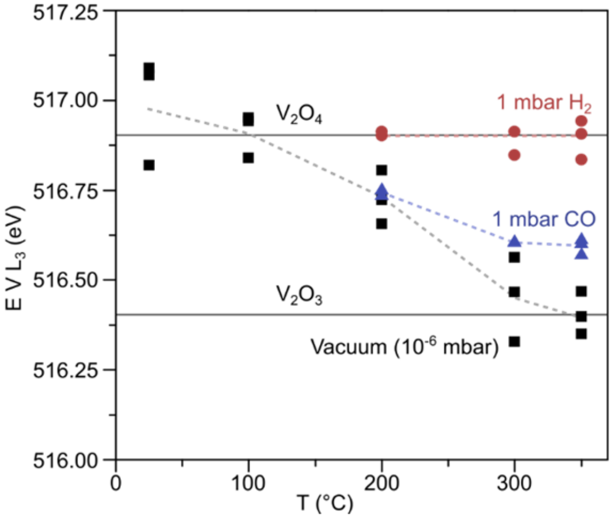

Supplementary Figure 7. Reduction of 2 monolayer VO_x/TiO_2 in different gaseous environments. Energy loss of the V L_3 peak for the 2 monolayer sample, measured by fitting a Gaussian function to the background-subtracted spectra, for increasing temperatures in three different reducing environments: 1 mbar H_2, 1 mbar CO, and 10^{-6} mbar vacuum. In H_2, the supported vanadium oxide is reduced only to V_2O_4, which is similar to the initial state in vacuum and room temperature. In CO, the oxide could be reduced further, but this environment provided no benefits over vacuum in terms of additional resistance to damage by the electron beam. Heating in vacuum was chosen as the reducing environment for the main investigation as it provided easy and reproducible access to the full range VO_x oxidation states.

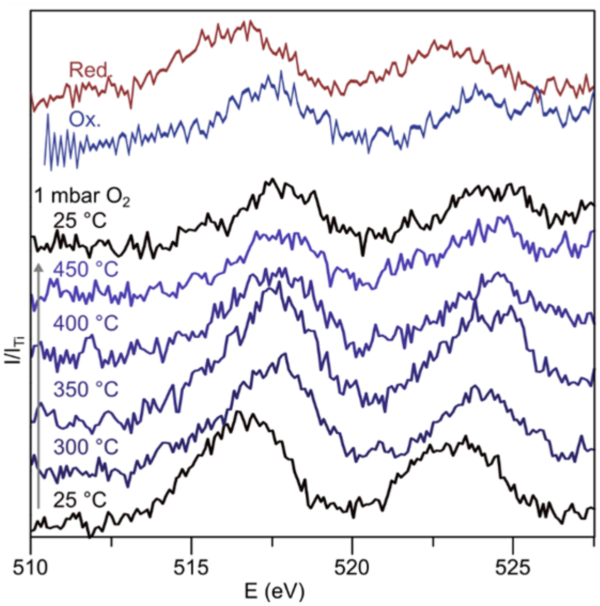

Supplementary Figure 8. EEL spectra showing the loss of vanadium during high temperature oxidation. EEL spectra recorded from a micrometre-sized area of a VO_x/TiO_2 agglomerate at increasing temperatures in a 1 mbar O_2 atmosphere. All spectra are shown after subtracting a power-law background and have been normalized so that the Ti L_2 peak has a peak intensity of 1 and have been vertically offset according to the order in which they were recorded. The loss of vanadium above 400 °C can be seen from the reduced height of the V $L_{2,3}$ peaks. The vanadium could not be recovered during subsequent reduction/oxidation cycles, as shown by the spectrum from the cooled sample. The remaining vanadium oxide exhibited reproducible shifts of the V L_3 peak similar to the original 2 monolayer sample before the high temperature oxidation as shown by the topmost two spectra acquired under first oxidizing (1 mbar O_2, 300 °C) and then reducing (10^{-6} mbar, 300 °C) conditions from the same area.

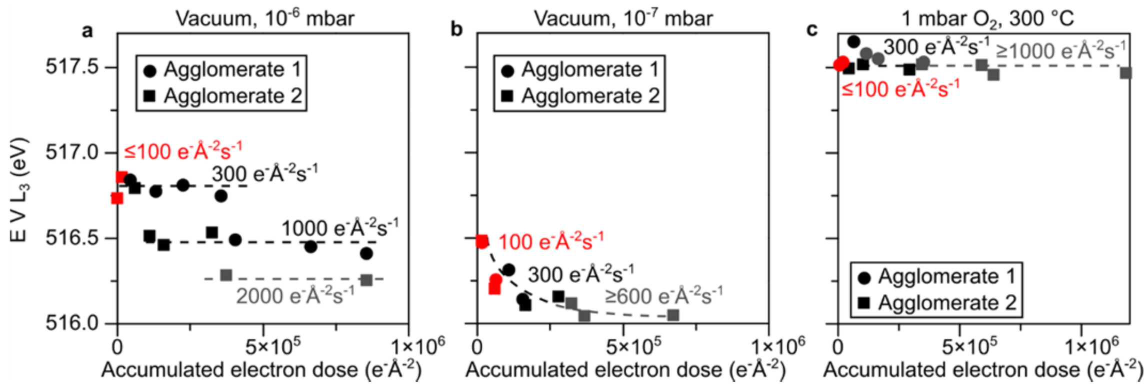

Supplementary Figure 9. Influence of the electron dose on the supported vanadium oxide. Energy loss of the V L_3 peak, measured by fitting a Gaussian function to the background-subtracted spectra, for increasing accumulated electron doses at room temperature and (**a**) 10^{-6} mbar vacuum (neutral condition), (**b**) 10^{-7} mbar dry vacuum, obtained by employing a liquid nitrogen cold trap, and (**c**) in 1 mbar O_2 at 300 °C. For each condition, measurements from two different agglomerates of VO_x/TiO_2 particles are shown. The measurements were performed also under varying electron dose rates, 30-2000 $e^-Å^{-2}s^{-1}$, which are noted next to the corresponding data-points. The measurements at the lowest accumulated electron doses have lower signal than the rest, making the peak position determination less precise. Under the neutral conditions (**a**), it is mainly the electron dose rate, rather than the accumulated electron dose, which determined the extent of the beam-induced reduction. Up to approximately 300 $e^-Å^{-2}s^{-1}$, no shift in the V L_3 peak could be detected, which indicates that the vanadium oxide was not affected by the electron beam to any measurable extent at this electron dose rate. In contrast, when employing the cold trap (**b**) the vanadium oxide was reduced even at very low electron dose rates, and the extent of the damage was instead mainly related to the accumulated electron dose. This finding resembles the gradual build-up of damage observed in the UHV STEM, but the difference in acceleration voltage and electron dose rate results in different structural effects (cf. Supplementary Fig. 20). In the oxidizing atmosphere of 1 mbar O_2 and 300 °C (**c**), no shift could be detected even at high electron dose rates and accumulated doses. The dashed lines serve to highlight the trend within each group of measurements at the same electron dose rate. At the reducing conditions (10^{-6} mbar vacuum, 300 °C), the beam-induced reduction proceeded to a similar extent as at the neutral condition, with V L_3 energies of 516.1-516.2 eV measured at 2000 $e^-Å^{-2}s^{-1}$.

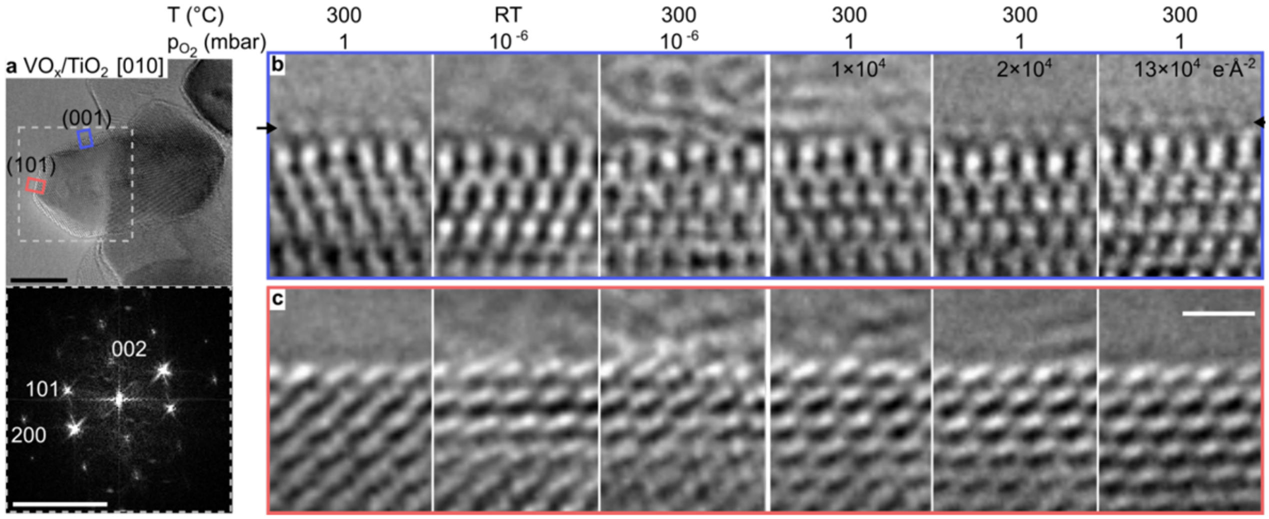

Supplementary Figure 10. TEM images acquired under continuous exposure to the electron beam. (**a**) Overview time-averaged image and corresponding FFT from a VO_x/TiO2 particle (the same as in Supplementary Figs. 12 and 13) indicating the areas from the (001) and (101) facets shown in more detail in **b** and **c**, respectively. Scale bar, 10 nm. The FFT is indexed according to the anatase [010] zone axis, showing the 2.10 nm^{-1} (002) and the 2.84 nm^{-1} (101) reflections, which were used for determining the orientation and faceting of the particle (scale bar, 5 nm^{-1}). The 20 individual images in the series were acquired with 2 s exposure times at an electron dose rate of 300 $e^-Å^{-2}s^{-1}$. See Supplementary Methods for a detailed description of the imaging method. The first three images in **b** and **c** were recorded under different oxygen pressures and temperatures. The last three images in **b** and **c** were all recorded under oxidizing conditions (300 °C, 1 mbar O_2) while continuously illuminating the sample with 300 $e^-Å^{-2}s^{-1}$ (the total dose during this exposure is noted for each image in **b**). The outermost layer on the (001) facet in **b**, only visible during the oxidizing conditions, is marked with two arrows. Note that no visible electron beam damage occurred during this series of images, as the electron dose rate was kept below the limit noted in Supplementary Fig. 9 at all times. Scale bar for **b** and **c**, 1 nm.

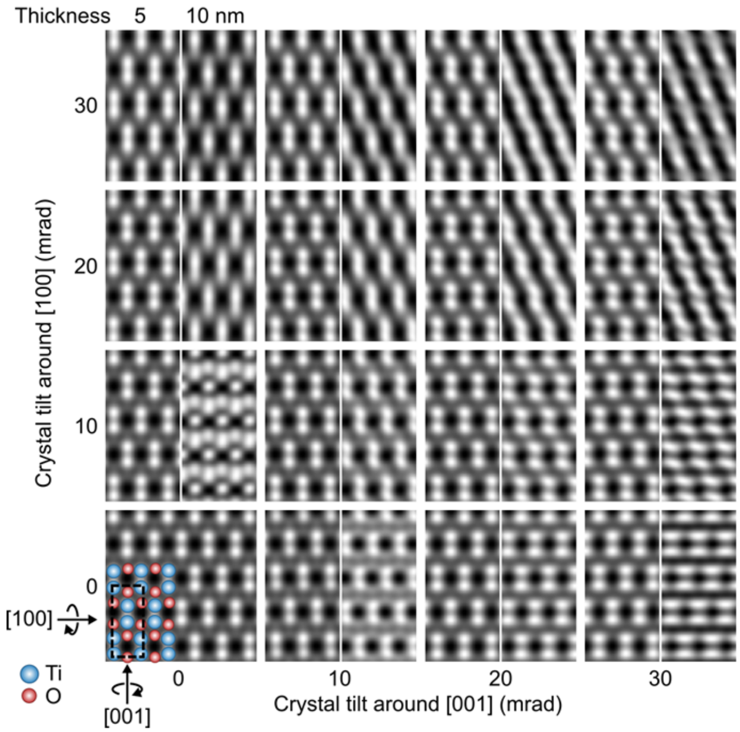

Supplementary Figure 11. Exit wave (EW) phase images simulated for tilted anatase crystals. Phase images were simulated for anatase TiO_2 tilted with respect to the [010] projection direction, at two thicknesses (5 and 10 nm), to facilitate comparison with the experimental images (see Supplementary Methods). The greyscale has been adjusted for each simulated EW phase image individually to cover the full range from black to white in order to highlight the contrast pattern. By comparison, figure 4a shows "bands" along the (101) planes, thus indicating a tilt of around 20/20 to 30/20 mrad in the [100]/[001] direction. The particles in figure 4b-c, as well as Supplementary Fig. 13, show no (or very faint) (101) "bands", indicating tilts of 10/10 mrads or less. In particular, the particle of figure 4c shows a contrast reversal in the thicker parts which can only be seen in the tilt map at 10/0 mrads. Supplementary Figs. 14, 15a, and 16 show less pronounced "bands" along (101), especially at the surface. Compared to the nanoparticle in figure 4a, this indicates small tilts. The particle in Supplementary Fig. 18 has an even smaller tilt, indicated by the fact that the (101) "bands" only appear further into the particle (i.e. at larger thicknesses). In general, the distortions in the contrast become less pronounced at the surface of the imaged nanoparticles, consistent with the simulated tilt map presented here. Most importantly, none of the tilts found are sufficient to distort the basic connection between the phase maxima and cation positions at the surface, and so will not affect the analysis presented in the main text.

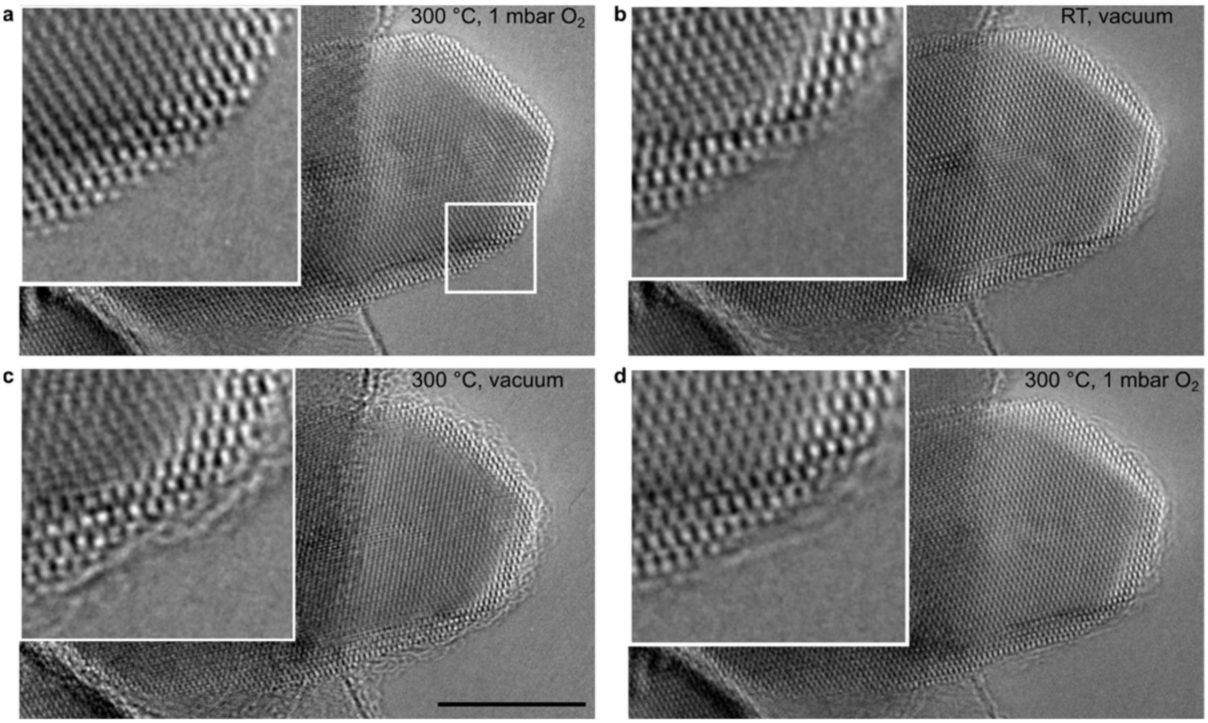

Supplementary Figure 12. TEM images of a VO_x/TiO_2 particle during reduction/oxidation. Time-averaged images recorded under (**a**) oxidizing (300 °C, 1 mbar O_2), (**b**) neutral (room temperature, vacuum), (**c**) reducing (300 °C, vacuum), and again (**d**) oxidizing conditions. The 20 individual images in the series were acquired with 2 s exposure times at an electron dose rate of 300 $e^-Å^{-2}s^{-1}$. See Supplementary Methods for a detailed description of the imaging method. The whole particle is shown in order to demonstrate that the particle maintained its overall shape during the changes in temperature and atmosphere. The insets show the corner between the (001) and (101) facet (from the same image) in more detail. Details from the surfaces are shown in Supplementary Fig. 13. The amorphous layer seen most clearly in **c** started to form already in **b**, but gradually disappeared under the oxidizing conditions shown in **d**. As there is no difference in the crystalline part between **b** and **c**, the amorphous layer cannot have formed from either the vanadium oxide or the titanium oxide, and must stem from contaminants in the microscope vacuum or from the MEMS device. The formation of such amorphous layers usually subsided after the first reduction/oxidation cycle. Scale bar, 10 nm.

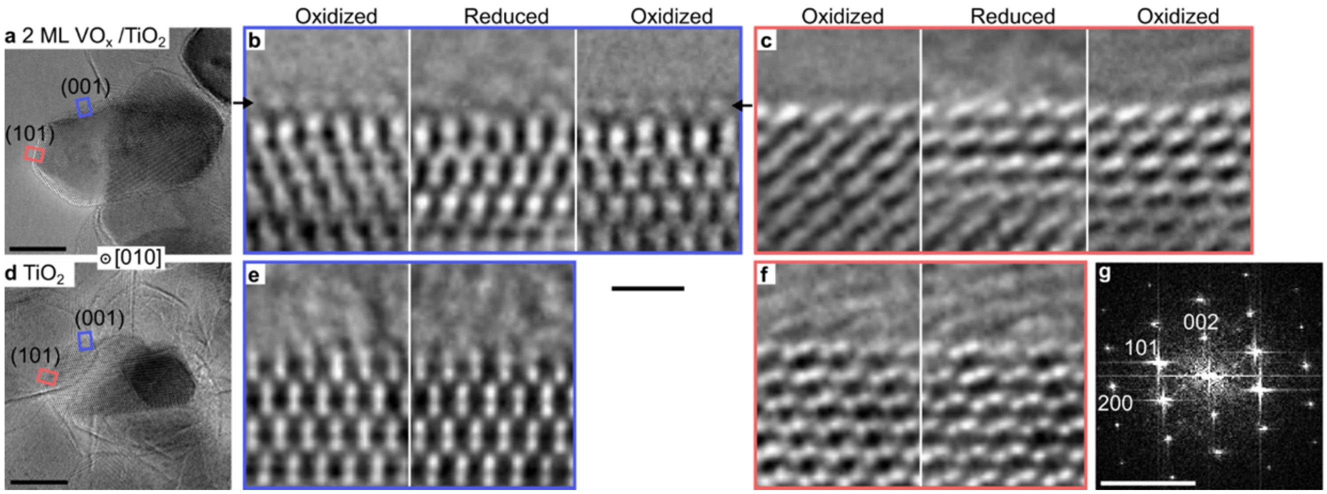

Supplementary Figure 13. Additional TEM images from a 2 monolayer VO_x/TiO_2 and a TiO_2 particle. (**a**) Overview TEM image of a 2 monolayer (ML) VO_x/TiO_2 particle (the same as in Supplementary Figs. 10 and 12) where the areas on the (001) and (101) facets shown in detail in **b** and **c**, respectively, are indicated. The outermost layer on the (001) facet, indicated by arrows, exhibited a periodic contrast feature isostructural to the anatase support under oxidizing conditions (300 °C, 1 mbar O_2) and a reduced and smeared contrast under reducing conditions (300 °C, vacuum). (**d**) Overview image of a pure TiO_2 particle where the areas on the (001) and (101) facets shown in detail in **e** and **f**, respectively, are indicated. The 20 individual images in the series were acquired with 2 s exposure times at an electron dose rate of 300 $e^{-}Å^{-2}s^{-1}$. See Supplementary Methods for a detailed description of the imaging method. Scale bars, 10 and 1 nm for overview and detail images, respectively. (**g**) FFT of the image in **d**, indexed according to the anatase [010] zone axis and showing the 2.10 nm^{-1} (002) and the 2.84 nm^{-1} (101) reflections used for determining the orientation and faceting of the particle (scale bar, 5 nm^{-1}).

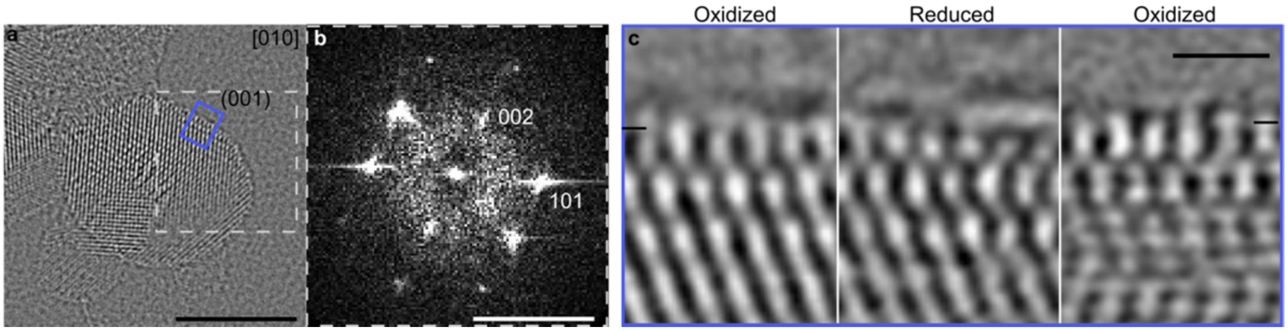

Supplementary Figure 14. Additional images from a 2 monolayer VO_x/TiO_2 particle. (**a**) Overview exit-wave (EW) phase image. Scale bar, 10 nm. (**b**) FFT of the area marked with a dashed box in image in **a**, indexed according to the anatase zone axis and showing the 2.10 nm^{-1} (002) and the 2.84 nm^{-1} (101) reflections used for determining the orientation and faceting of the particle (scale bar, 5 nm^{-1}). The small area marked at the (001) facet is shown in detail in **c**, as the environment in the microscope was changed from oxidizing (300 °C, 1 mbar O_2), to reducing (300 °C, vacuum) and back to oxidizing again. The particle was tilted slightly away from the [010] direction, resulting in a loss of resolution at the (101) facet which is therefore not shown at high magnification. Scale bar, 1 nm.

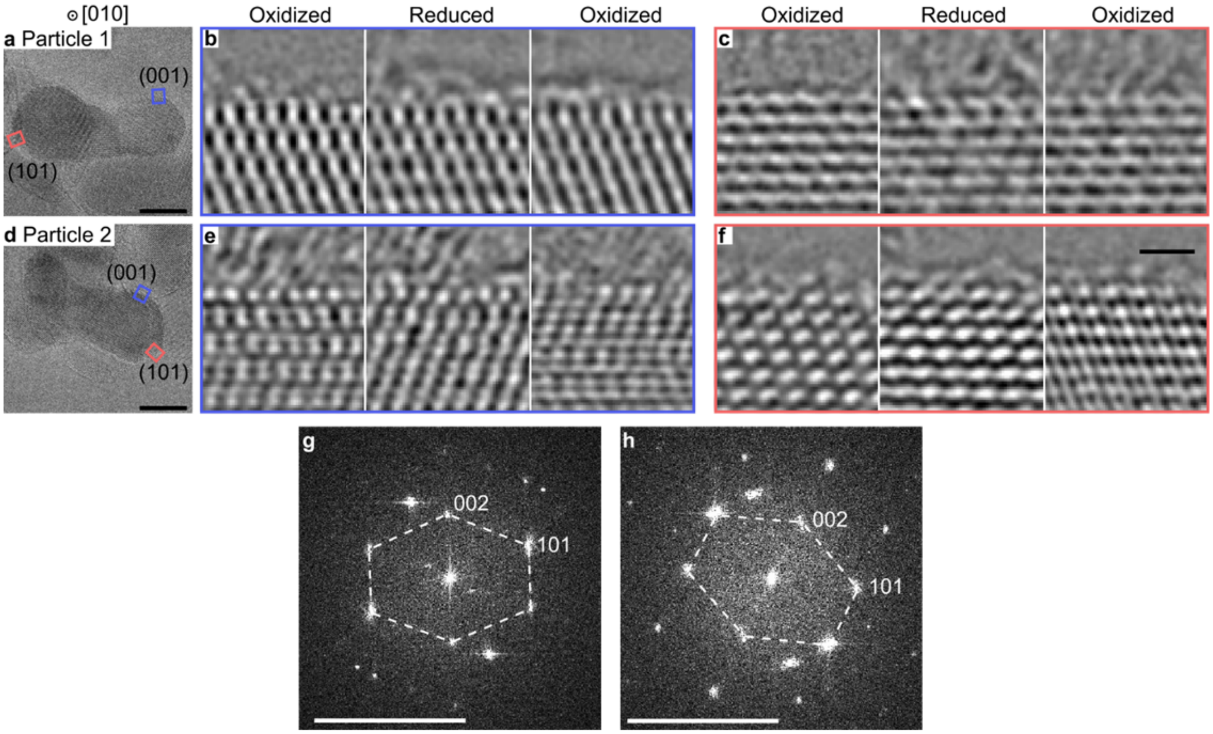

Supplementary Figure 15. Additional images from 0.5 monolayer VO_x/TiO_2 particles. (**a**, **d**) Overview TEM images of two 0.5 monolayer VO_x/TiO_2 particles with areas marked on the (001) and (101) facets, indicating where the EW phase images were cropped for the subsequent panels (**b**, **e** and **c**, **f**, respectively) as the environment in the microscope was changed from oxidizing (300 °C, 1 mbar O_2) to reducing (300 °C, vacuum), and back to oxidizing again. Scale bars, 10 and 1 nm for overview and detail images, respectively. **g** and **h** show FFTs of the images in **a** and **d**, respectively. Both have been indexed according to the anatase [010] zone axis and show the 2.10 nm^{-1} (002) and the 2.84 nm^{-1} (101) reflections used for determining the orientation and faceting of the particles (scale bars, 5 nm^{-1}). The dashed line serves to highlight the reflections from the imaged particle with respects to reflections stemming from surrounding particles.

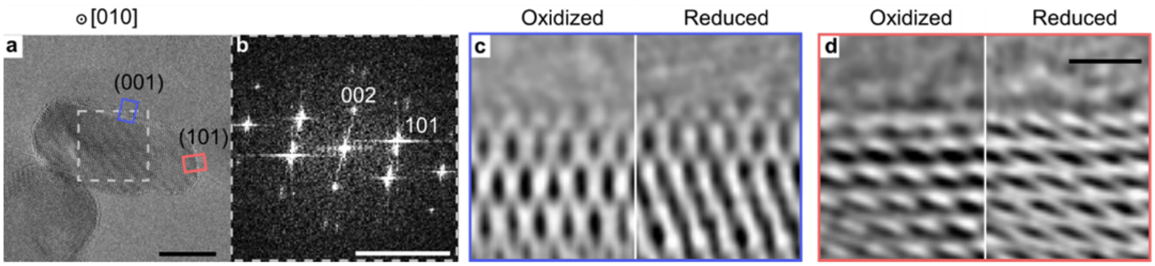

Supplementary Figure 16. Additional images from a pure TiO_2 particle. (**a**) Overview TEM images of a pure TiO_2 particle with areas marked on the (001) and (101) facets, indicating where the EW phase images were cropped for the subsequent panels (**c** and **d**) as the environment in the microscope was changed from oxidizing (300 °C, 1 mbar O_2) to reducing (300 °C, vacuum). Scale bars, 10 and 1 nm for overview and detail images, respectively. (**b**) FFT of the area marked with a dashed box in image **a**, indexed according to the anatase [010] zone axis and showing the 2.10 nm^{-1} (002) and the 2.84 nm^{-1} (101) reflections used for determining the orientation and faceting of the particle (scale bar, 5 nm^{-1}).

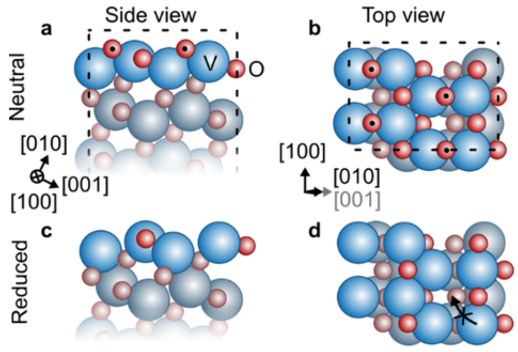

Supplementary Figure 17. Structure and DFT analysis of the reduced VO_x (101) surface. Structure of a two-layer VO_2 phase attached to the (101) surface of a four-layer thick anatase slab, viewed (**a**) perpendicular and (**b**) normal to the surface. In the top views (**b** and **d**) only the top two layers are shown. Reduction was modelled by removing the two-fold coordinated oxygen atoms marked with black dots, yielding a $VO_{1.5}$ composition for the outermost layer. The reduced phase is shown (**c**) perpendicular and (**d**) normal to the surface. At the reduced (101) surface, the vanadium atoms with the lowest coordination have four neighbouring oxygen atoms in contrast to only three neighbouring oxygen atoms at the correspondingly reduced (001) surface. The lower under-coordination explains why the V atoms within the top layer of the (101) surface undergo only small displacements (of the order of a few tens of picometres) to accommodate the loss of oxygen and why all atoms essentially retain their original positions. Consequently, these changes have not yet been detected in the TEM images, e.g. in figure 4a in the main text. Further displacement of the surface vanadium atoms, as indicated by the arrow in **d**, did not decrease the energy, and there is no driving force for a substantial reconstruction of the reduced surface. Furthermore, the higher density of vanadium atoms on the (101) surface (10 atoms/nm^2) compared to the (001) surface (7 atoms/nm^2) will lead to a higher barrier for surface diffusion.

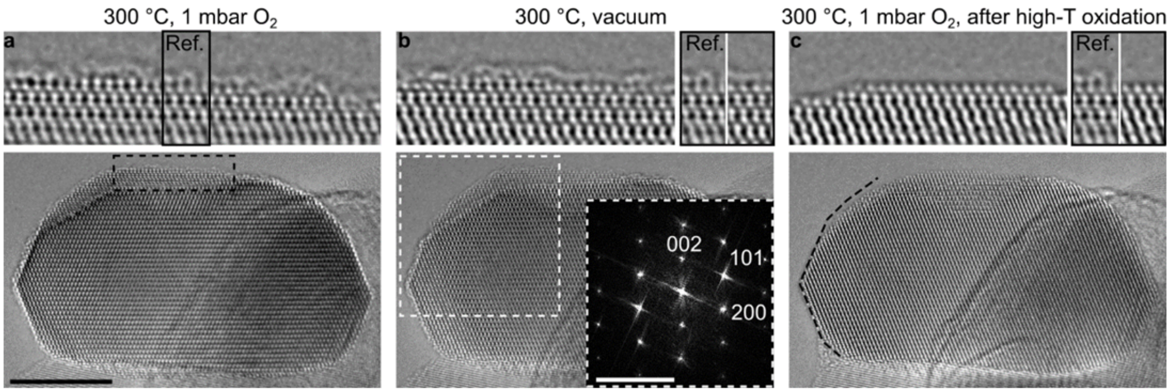

Supplementary Figure 18. Structural changes in VO_x/TiO_2 surface layer after high temperature oxidation. (**a**) The lower frame shows an overview time-averaged TEM image (20 images, 2 s exposures, 300 $e^-Å^{-2}s^{-1}$) showing a single VO_x/TiO_2 particle in a [010] orientation under oxidizing conditions (300 °C, 1 mbar O_2). Scale bar, 10 nm. The upper frame shows a detail, corresponding to the area indicated in the overview image, cropped from an EW phase image acquired immediately after the TEM overview image. (**b**) Corresponding images acquired under reducing conditions (300 °C, vacuum). Note that the top crystalline layer has been removed and instead the particle is surrounded by an amorphous layer. This is most clearly seen in the inset where the reference area marked in **a** is shown next to the identical area from **b**. The inset shows an FFT (calculated from the area indicated in the overview image) indexed according to the anatase [010] zone axis and showing the 2.10 nm^{-1} (002) and the 2.84 nm^{-1} (101) reflections used for determining the orientation and faceting of the particle (scale bar, 5 nm^{-1}). (**c**) Corresponding images acquired under oxidizing conditions (300 °C, 1 mbar O_2) after heating the sample to 550 °C in 5 mbar O_2. The high temperature oxidation resulted in an irreversible loss of the outermost crystalline layer and was associated with a reduction of the vanadium EEL signal intensity (see figure 3 in the main text and Supplementary Fig. 8). During the high temperature oxidation, the morphology of the particle at the (101) facets also changed, as denoted by the dashed line which traces the outline of original particle shape from **a**. The morphology change makes detailed analysis of the surface transformation difficult, as one cannot be certain that the exact same areas are compared in the before and after images.

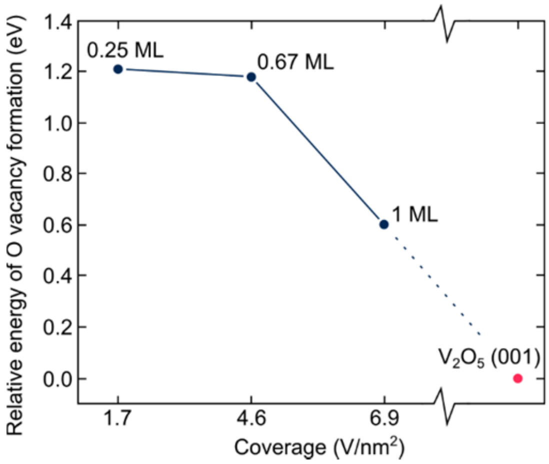

Supplementary Figure 19. Trend in reducibility of $VO_{2.5}/TiO_2$ at coverages below a single monolayer. The calculated formation energy for a single oxygen vacancy in $VO_{2.5}$ phases at various coverages on a TiO_2 (001) surface is shown relative to the corresponding energy for an unsupported V_2O_5 (001) slab. The cost of creating an oxygen vacancy was found to increase with decreasing coverage. This trend continues that reported by Vittadini et al. in the range of 1-3 epitaxial monolayers of VO_x on TiO_2.[9] As a general rule, reduced VO_x surface phases are more easily oxidized (or conversely, $VO_{2.5}$ phases are more difficult to reduce) the lower the coverage is on the TiO_2 surface. Below about 0.67 monolayers, the reducibility was found to vary much less, as the vanadium atoms at these lower coverages interact to a much lesser degree through e.g. bridging oxygen atoms. The trend presented here agrees with the previously reported finding that the two outermost layers of bulk V_2O_5 are transformed to VO during thermal reduction,[10] while a reduction beyond $VO_{1.5}$ was never measured for the supported VO_x phases. In the calculations, the VO_x/TiO_2 (001) was represented by three layers of TiO_2. The bottom layer was fixed while the rest were relaxed. For the 1.7 V/nm^2 coverage the structure was a monomeric VO_3H in a 2x2 supercell. For the 4.6 V/nm^2 and 6.9 V/nm^2 coverages, we cut a V_4O_{10} cluster from a V_2O_5 (001) slab, aligned it to the TiO_2 (001) surface, and optimized the structure in 3x3 and 3x2 supercells respectively. The DFT methodology is detailed in the main paper.

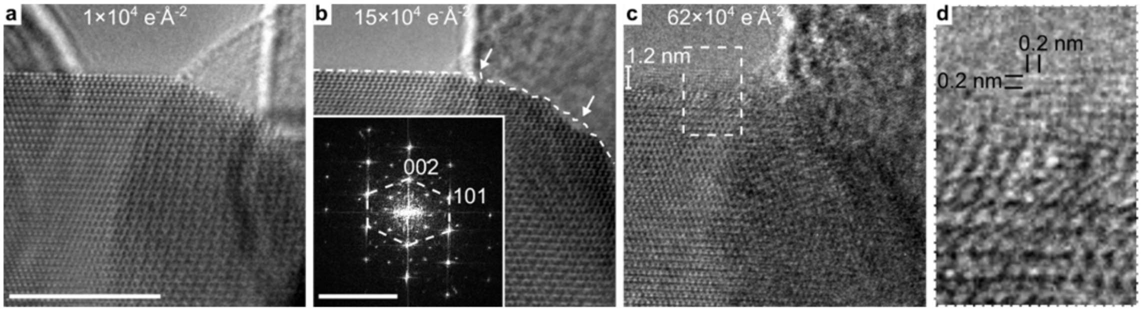

Supplementary Figure 20. TEM images acquired while employing the liquid nitrogen cold trap. TEM images showing the corner between a (001) and (101) facet on a VO_x/TiO_2 particle, recorded under a 10^{-7} mbar vacuum employing the liquid nitrogen cold trap at: (**a**) 300 °C and (**b–d**) room temperature (scale bar, 10 nm). The accumulated electron dose is noted at the top of each image. Note the absence of an amorphous layer. The outline of the particle in **a** has been superimposed on the image in **b** in order to highlight where material has been irreversibly removed by the electron beam, even though the electron dose rate was limited to 300 $e^{-}Å^{-2}s^{-1}$. With such clear differences in the overall shape, it is difficult to align the images accurately enough to detect any subtle changes, e.g. in the outermost layer on the (001) facet. The inset in **b** shows the corresponding FFT indexed according to the anatase [010] zone axis, showing the 2.10 nm^{-1} (002) and the 2.84 nm^{-1} (101) reflections used for determining the orientation and faceting of the particle (scale bar 5 nm^{-1}). The dashed line serves to highlight the reflections from the particle against the background stemming from a Moiré pattern where two particles overlap. After approximately 8 minutes of exposure to the electron beam at a dose rate of 1000 $e^{-}Å^{-2}s^{-1}$, a roughly 1 nm thick reduced surface layer was formed (**c**). The area marked by the dashed box is shown in the close-up in **d**. The surface layer consisted of a cubic phase with a 0.2 nm lattice spacing, which matches the (002) plane spacing of either TiO or VO well. Likely, both the V-oxide layer and a few of the outermost layers of the TiO_2 particle have been reduced by the electron beam as the reduced phase is too thick to stem only from the V-oxide. Images **a** and **b** were time-averged from 20 individual images acquired with 2 s exposure times at an electron dose rate of 300 $e^{-}Å^{-2}s^{-1}$. See Supplementary Methods for a detailed description of the imaging method. For **c** and **d**, a single exposure image acquired at an electron dose rate of 1000 $e^{-}Å^{-2}s^{-1}$, is shown.

SUPPLEMENTARY NOTE 1

The origin of the amorphous layer occasionally observed at reducing conditions

Amorphous layers were sometimes observed to form in the reduced state (most clearly seen on (101) in figure 4b). Such layers are, however, not necessarily related to the mobile vanadium atoms discussed in the main text as they sometimes formed also on the 0.5 monolayer VO_x/TiO_2 and pure TiO_2 samples, without a corresponding loss of crystalline layers. In these cases, the layers most likely stem from contaminants adsorbing on the particle surfaces. The layers formed in this fashion can easily be distinguished from the disordered VO_x discussed in the man text: the contamination layers form on top of the crystalline material, while the disordered VO_x forms from, and replaces, the outermost crystalline layer. Therefore, the occasional presence of contamination layers does not affect the conclusions presented in the main text.

A further indication that these layers are not inherent to the sample itself is that they did not form when employing a liquid nitrogen cold trap to reduce the background pressure in the *in situ* TEM sample chamber (see Supplementary Fig. 20). At this lower pressure the sensitivity of the VO_x/TiO_2 particles to the electron beam increased dramatically. Consequently, all images were acquired without use of the cold trap, with the exception of Supplementary Fig. 20. This is described in more detail in connection with Supplementary Figs. 9 and 20. Interestingly, only when employing the cold trap was it possible to form the beam-induced, rock-salt structured VO and TiO surface layers which have often been reported in previous TEM studies of VO_x/TiO_2.[5–7] However, such large structural changes also precluded accurate alignment of the images, and therefore also detailed structural comparisons with the pristine particles.

SUPPLEMENTARY METHODS

Image acquisition and analysis

Electron beam damage imposed a strict limit on the electron dose rate throughout the *in situ* TEM experiments, as discussed in more detail in Supplementary Figs. 9, 10, 12 and 20. Single shot images therefore had too low signal-to-noise ratio for studying the details of the particle surfaces. Two imaging strategies were used to enhance the image signal: exit-wave (EW) reconstruction from focal series and averaging of time series acquired at a single defocus value. The former has the advantage of allowing post-acquisition correction of remaining aberrations, but its data processing is more time consuming. Both types of image series were typically recorded at 1024×1024 pixels with a pixel size of 0.048 nm/pixel, covering the entire particles.

The time-averaged image series each comprise 10-20 images acquired with an overfocus of a few nanometres to compensate the spherical aberration, which had been tuned to approximately -15 μm. The individual images were (1) visually inspected to make sure that the particle remained unchanged in the successive images, (2) aligned to sub-pixel accuracy using phase correlation, and (3) finally summed.

The focal series each comprise 35 images spanning a defocus range of approximately +40 to -30 nm with a nominal 2 nm defocus step. EW reconstructions were performed using the Gerchberg-Saxton algorithm,[11] as implemented in the MacTempas software (www.totalresolution.com). After the initial restoration, the defocus and two-fold astigmatism were further optimized in order to yield as large phase variation and as little amplitude variation as possible in the resulting EW images.

Following the processing of the image series, the resulting summed images or exit wave images have been cropped to various degrees for the figures in the main text and the supplementary. The main text focuses on the EW phase images as the contrast in these is more directly relatable to the position and composition of the atomic columns.

The orientation and crystal structure of each particle was verified from the high resolution images and corresponding FFT. In the anatase [010] viewing direction, the 2.84 nm^{-1} (101) and 2.10 nm^{-1} (002) reflections provide a unique pattern that does not occur in the other TiO_2 polymorphs, i.e. rutile, brookite, or TiO_2-B.

Image simulation

In order to relate the patterns in the reconstructed EW images to the atomic structure, multi-slice simulations were performed using MacTempas. A model was constructed of an anatase TiO_2 particle using the assumption that the particles were bound only by {001} and {101} facets. Using this model, images were simulated for an electron microscope operated at 300 keV primary electron energy and with a nominal 3.5 nm defocus spread. A 0.1 nm mechanical vibration was added to best match the experimental EW phase images. The simulated EW phase, as shown in the inset in figure 4 in the main text, indicates that the bright spots are related to the metal atom positions, while the O positions are visible only as a smooth grey band. The intensities in the experimental and simulated images are not shown on the same scale, and should be used for qualitative comparisons only. Substitution of Ti for V in the model produced no visible difference in the EW phase image.

The effect of tilt of the anatase crystal with respect to the [010] projection direction was examined by shifting the centre of the Laue circle towards the (002) and (200) reflections in the simulations (the tilts in the opposite directions are equivalent due to symmetry). These simulations, shown in Supplementary Fig. 11, were performed using the JEMS software (4.4431U2016) and a slab TiO_2 model, but otherwise used the imaging conditions described above.

For anatase at 5 nm thickness, the contrast pattern remains directly related to the anatase cation positions with minimal distortions up to 30 mrad (1.7°) tilt in either direction. For anatase at 10 nm thickness, there is a contrast reversal at very small tilts, as evidenced by the prominent contrast of the O positions and the vacant space between adjacent cation columns at 10/0 mrad [100]/[001] tilt. As the corresponding contrast pattern is inverted, it is clearly distinct from the others and therefore eliminates an erroneous assignment of cation to O positions. The phase maxima return to the cation columns and the anatase cation structure is again directly discernible at increasing tilts of the 0-20/10-30 mrad [100]/[001] tilt range. At low tilts around [001] and higher tilts around [100], i.e. 20-30/0-10 mrad [100]/[001] tilts, the two adjacent cation positions produce a single elongated feature in the phase image. At large tilts in both directions, i.e. 30/20-30 mrad [100]/[001] tilts, the two adjacent cation positions are also resolved. As the phase maxima are extended in the tilt direction, “bands” form along the (101) planes at these tilts. In summary, the majority of the tilt direction/magnitudes show phase maxima directly

related to the cation positions, making the anatase structure directly discernible from the pattern. Thus, the analysis of cation structure from phase images is robust in regards to tilt effects.

SUPPLEMENTARY REFERENCES

1. Wachs, I. E. Selective catalytic reduction of NO with NH_3 over supported vanadia catalysts. *J. Catal.* **161,** 211–221 (1996).

2. Nitsche, D. & Hess, C. Normal mode analysis of silica-supported vanadium oxide catalysts: Comparison of theory with experiment. *Cat. Commun.* **52,** 40–44 (2014).

3. Brázdová, V., Ganduglia-Pirovano, M. V. & Sauer, J. Crystal structure and vibrational spectra of $AlVO_4$. A DFT study. *J. Phys. Chem. B* **109,** 394–400 (2005).

4. McCartney, M. R. & Smith, D. J. Epitaxial relationships in electron-stimulated desorption processes at transition metal oxide surfaces. *Surf. Sci.* **221,** 214–232 (1989).

5. Wallenberg, L. R., Sanati, M. & Andersson, A. A high-resolution electron microscopy investigation of TiO_2(B)-supported vanadium oxide catalysts. *J. Catal.* **126,** 246–260 (1990).

6. Srinivasan, S. & Datye, A. K. Transmission electron microscopy of supported molybdenum and vanadium oxides. *Catal. Letters* **15,** 155–167 (1992).

7. Nobbenhuis, M. G., Wessicken, R., Probst, W., Mallat, T. & Baiker, A. Study of the morphology of vanadia / titania and tin-oxide-promoted vanadia / titania catalysts by electron microscopic methods. *Appl. Surf. Sci.* **78,** 99–106 (1994).

8. Su, D. S. *et al.* Electron beam induced reduction of V_2O_5 studied by analytical electron microscopy. *Catal. Letters* **75,** 81–86 (2001).

9. Vittadini, A., Casarin, M., Sambi, M. & Selloni, A. First-Principles Studies of Vanadia - Titania Catalysts: Beyond the Monolayer. *J. Phys. Chem. B* **109,** 21766–21771 (2005).

10. Devriendt, K., Poelman, H. & Fiermans, L. Thermal reduction of vanadium pentoxide: an XPD study. *Surf. Sci.* **433–435**, 734–739 (1999)

11. Gerchberg, R. W. & Saxton, W. O. A practical algorithm for the determination of phase from image and diffraction plane pictures. *Optik* **35,** 237–246 (1972).
